# Probing flow-induced nanostructure of complex fluids in arbitrary 2D flows using a fluidic four-roll mill (FFoRM)

**DOI:** 10.1038/s41598-018-33514-8

**Published:** 2018-10-22

**Authors:** Patrick T. Corona, Nino Ruocco, Kathleen M. Weigandt, L. Gary Leal, Matthew E. Helgeson

**Affiliations:** 1 University of California Santa Barbara, Santa Barbara, CA 93106-5080 USA; 2 Present Address: ExxonMobil Chemical Company, Baytown Technology and Engineering Complex, Baytown, TX 77520 USA; 3 National Institute of Standards and Technology, Center for Neutron Research, Gaithersburg, MD 20899-6102 USA

## Abstract

Engineering flow processes to direct the microscopic structure of soft materials represents a growing area of materials research. *In situ* small-angle neutron scattering under flow (flow-SANS) is an attractive probe of fluid microstructure under simulated processing conditions, but current capabilities require many different sample environments to fully interrogate the deformations a fluid experiences in a realistic processing flow. Inspired by recent advances in microfluidics, we present a fluidic four-roll mill (FFoRM) capable of producing tunable 2D flow fields for *in situ* SANS measurements, that is intended to allow characterization of complex fluid nanostructure under arbitrary complex flows within a single sample environment. Computational fluid dynamics simulations are used to design a FFoRM that produces spatially homogeneous and sufficiently strong deformation fields. Particle tracking velocimetry experiments are then used to characterize the flows produced in the FFoRM for several classes of non-Newtonian fluids. Finally, a putative FFoRM-SANS workflow is demonstrated and validated through the characterization of flow-induced orientation in a semi-dilute cellulose nanocrystal dispersion under a range of 2D deformations. These novel experiments confirm that, for steady state straining flows at moderate strain rates, the nanocrystals orient along the principal strain-rate axis, in agreement with theories for rigid, rod-like Brownian particles in a homogeneous flow.

## Introduction

The coupling of soft material microstructure with complex flows – involving deformations other than pure elongation or viscometric (shear) flow – plays a crucial role in a variety of industrial processes including extrusion, fiber spinning, injection or blow molding, and various coating flows. New applications for soft materials have highlighted the need for flow-processed materials with specific and highly-ordered or oriented microstructure to achieve superior mechanical, optical or transport properties. Examples include shape changing composites, polymer opals, and filtration membranes^1–4^. Most of these materials and their precursors exhibit complex, nonlinear rheological responses (*e.g*. shear thinning, shear/extensional thickening, strain hardening, or yield stresses) due to strong coupling of their microstructural order with flow. As such, although the desired microstructure of a processed material may be known, the optimal flow protocol, *i.e*., the types and rates of deformation that the material should be subjected to in order to achieve the target microstructure is not always obvious. At the same time, many of these emerging materials lack accurate, microstructurally-informed rheological models that would enable inverse design of flow processes for soft materials. Therefore, an enormous advantage would be gained by the development of methods for experimentally characterizing the evolution of fluid microstructure under arbitrarily complex flows.

Small angle neutron scattering (SANS) provides an especially attractive platform for developing such microstructure—property-processing relationships. Unlike other types of radiation, neutrons are passive probes of fluid microstructure at length scales important to most soft materials (1–1000 nm). Furthermore, neutron contrast can be easily varied with isotopic substitution, enabling independent interrogation of the components of mixtures that is not possible with other techniques. This has led to the recent development of a number of flow devices compatible with *in situ* neutron scattering measurements (flow-SANS), allowing direct measurement of fluid microstructure under flow^5,6^. A recent review outlines the most extensively used flow-SANS sample environments, including Couette geometries, pressure driven Poiseuille flows, capillary flow cells, sliding plate cells, and cross-slot devices^5^. With a few exceptions such as the cross-slot device, these systems create a pure shear deformation of the fluid. Thus, the creation of a flow-SANS sample environment to achieve tunable deformation fields would dramatically expand the range and usefulness of flow-SANS measurements under complex flows that approach more realistic processing flows.

Recently, there have been a number of efforts toward this aim, typically involving the use of fluidic devices that simulate a processing flow of interest. Examples include channel flows^7–9^, cross slot geometries to achieve relatively homogeneous 2D elongation^10–13^, contraction/expansion geometries to study axisymmetric entrance and exit flows^6,14–16^, and flows around obstacles^17^. These studies highlight a number of challenges and limitations inherent to the use of fluidic devices for flow-SANS measurements. The first, and most significant, is that the deformation field encountered by the fluid is determined predominately by the geometry of the device, and changes as the fluid traverses the geometry, producing an unsteady deformation of the fluid in a Lagrangian (material point) frame. Second, although many devices produce the desired flow at the center-plane, the aspect ratio (defined by the out-of-plane and in-plane dimensions relative to the neutron beam) is typically small, which produces a significant shearing contribution to the deformation along the beam path, similar to a Hele-Shaw type flow. This effect produces significant non-uniformity of the flow, and thus the fluid microstructure, in the direction of the probing beam path. Because of these limitations, the flow-SANS measurement reflects the material configuration that results from a non-steady, spatially non-uniform deformation field. As such, an accurate rheological model and corresponding flow simulations are required to properly interpret microstructural information obtained from these measurements. This significantly limits the applicability of current flow-SANS devices to only those fluids for which such models exist.

These limitations highlight a need for new designs for flow-SANS sample environments with the following objectives: (1) the deformation field can be varied (including mixtures of shear and elongation) within the same device to avoid construction of many different devices; (2) the deformation field is homogeneous within the scattering volume and in as large a region as possible around the scattering volume; (3) a stagnation point exists that can be positioned within the scattering volume. The latter two conditions are intended to ensure that the accumulated time or strain for material within the homogeneous flow region is sufficiently long so as to achieve a steady state response in the fluid microstructure.

A flow device has been previously developed, originally aimed at studying the deformation of a drop or vesicle, that satisfies these criteria, namely the four-roll mill. The most ideal form of this device was originally designed by Taylor^18^, in which the flow is driven by rotation of four cylinders. Although this configuration was used in the past for birefringence studies of polymer solutions^19,20^, it has not yet been successfully developed at the miniature scale required for SANS due to difficulty of scaling down the size of mechanical rollers (and therefore of the device itself), resulting in unacceptably large sample requirements and a beam path that results in a loss of transmitted signal and potential multiple scattering effects. There is also the practical difficulty of sealing the device from leaks when the rollers are oriented orthogonal to the direction of gravity (a requirement to facilitate measurement with most neutron beams).

An alternative approach is the flow-through design of Lee *et al*., which is a generalization of the cross-slot flow^21^. It is generally accepted that this “fluidic four-roll mill” (FFoRM) design holds the most promise as a flow device based upon the ideas of the four-roll mill, except that the flow is driven externally by syringe pumps similarly to the cross-slot and the only limit to size is the ability to manufacture the device via etching methods. Here, we report on the design and experimental validation of such a device, optimized to produce a homogeneous flow over a large area for the spectrum of flows from shear to 2D extension for Newtonian fluids. Furthermore, we explore to what extent the full spectrum of 2D homogeneous flows can be produced for a variety of non-Newtonian and/or viscoelastic fluids, and demonstrate the applicability of the full FFoRM-SANS workflow for dispersions of nano-crystalline rods that exhibit shear thinning, but only weakly elastic behavior, where the orientation measured by SANS provides a probe of the local deformations in the device.

## A Fluidic Four-Roll Mill (FFoRM) For SANS Measurements

Without loss of generality, one can describe any 2D linear flow field

1 $${\bf{u}}=\nabla {\bf{u}}\cdot {\bf{x}}=({\bf{E}}+{\boldsymbol{\Omega }})\cdot {\bf{x}}$$

by decomposing the local velocity gradient tensor into two parameters related to the deformation type and magnitude of the deformation rate at any material point in the flow^22^. Here **E** and **Ω** are the strain-rate and vorticity tensors. The flow type parameter is usually defined as

2 $$\Lambda =\frac{|{\bf{E}}|-|{\boldsymbol{\Omega }}|}{|{\bf{E}}|+|{\boldsymbol{\Omega }}|}$$

where |**E**| is the magnitude of the rate of strain tensor and |**Ω**| is that of the vorticity tensor (where $$|{\bf{A}}|={({\bf{A}}:{{\bf{A}}}^{{\boldsymbol{T}}})}^{1/2}$$$$\equiv {{\rm{A}}}_{{\boldsymbol{ij}}}{{{\rm{A}}}_{{\boldsymbol{ij}}}}^{1/2}$$). For the approximately 2D, planar flows considered in this work, the flow type parameter takes values from −1 (pure rotation) to 1 (pure elongation) and describes the relative magnitudes of vorticity and strain-rate present in the flow. The strength of the flow can be described through the magnitude of the in-plane components of the velocity gradient tensor ($$\dot{\Gamma }$$) defined as $$\dot{\Gamma }=|\nabla {\boldsymbol{u}}|$$. Hence, in 2D homogeneous flows the velocity gradient tensor can be expressed in terms of $$\dot{\Gamma }$$ and *Λ* as^23–25^

3 $$\nabla {\boldsymbol{u}}=\frac{\dot{\Gamma }}{2\sqrt{1+{\Lambda }^{2}}}[\begin{array}{ccc}1+\Lambda  & 1-\Lambda  & 0\\ -(1-\Lambda ) & -(1+\Lambda ) & 0\\ 0 & 0 & 0\end{array}]$$

with axes defined in the velocity (1) and velocity gradient (2) directions, hereafter referred to as the in-plane axes. Written in this form, $\frac{\dot{\Gamma }}{\sqrt{1+{\Lambda }^{2}}}$ is equivalent to the shear rate ($\dot{\gamma }$) for a simple shear flow (*Λ* = 0) and the extension rate ($\dot{\varepsilon }$) for the purely extensional planar flow (*Λ* = 1). The assumption that the flow is 2D, as assumed in equation (3), is approximately true in the microfluidic four-roll mill provided that the dimensions of the flow device in the out-of-plane direction are large enough that ▽***u*** is dominated by in-plane contributions for the majority of the flow domain. Specifically, we shall see that the 2D approximation, namely that *u*_3_ = 0, and $\frac{\partial {u}_{1}}{\partial {x}_{3}}=\frac{\partial {u}_{2}}{\partial {x}_{3}}=0$, is reasonable in the central region between the top and bottom boundaries (*u*_3_ = 0 at the central symmetry plane), and near the central stagnation point.

**Figure 1:**
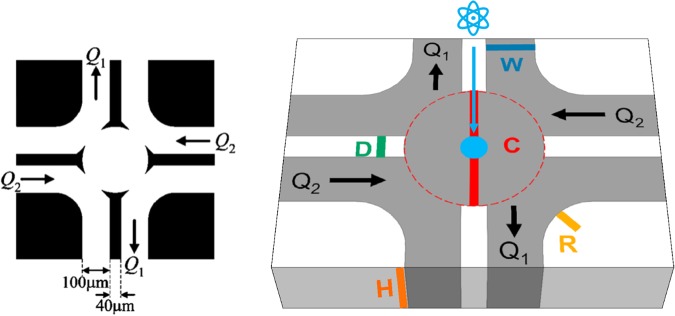
(left) Original microfluidic four-roll mill design reproduced from Lee *et al*.^21^. (right) Schematic 2D representation of the parameterized FFoRM geometry. Geometric parameters that were varied in the device design are indicated with colored letters. The 1 mm diameter neutron beam cross section is indicated as a light blue circle in the center of the device. The included values of these geometric parameters are the final choice of geometry with *W* = 2 mm, *D* = 1 mm, *R* = 1 mm, *C* = 5 mm, and *H* = 3 mm. Channels with controlled flow rates *Q*_1_ and *Q*_2_ are indicated with black arrows.

The original microfluidic four-roll mill is sketched in Fig. 1a. It is similar to a cross-slot flow device, but is composed of eight channels where the relative volumetric flow rates in diagonally opposing pairs of four channels control the flow type within the device, while the average of the flow rates in these four channels controls the flow strength. The remaining four channels are held at constant (usually ambient) pressure. Although Lee *et al*. showed that the device can produce a reasonable approximation of the full spectrum of homogeneous 2D flows for Newtonian fluids, it is not clear whether it will produce homogeneous 2D flows when the fluid is non-Newtonian and/or viscoelastic.

**Figure 2:**
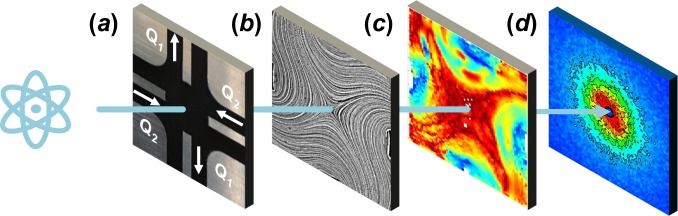
Schematic outline of FFoRM-SANS workflow. (**a**) The parameterized FFoRM geometry is adjusted to generate spatially uniform flows within design constraints. Flow field visualization enables the operational mapping of the (**b**) streamlines and (**c**) flow type/deformation rate field the fluid experiences. (**d**) Small-angle neutron scattering probes structural changes in the material under arbitrary deformation histories.

In this work (summarized in Fig. 2), we explore this question, as well as the applicability of the fluidic four-roll mill (FFoRM) for SANS measurements. In particular, we show that a modified version of the microfluidic geometry of Lee *et al*. can produce tunable 2D deformation fields for at least some non-Newtonian fluids, and can be integrated with SANS instrumentation for measurement of the *in situ* microstructural configuration projected onto the velocity-velocity gradient plane. CFD simulations were used to optimize the device geometry to produce flows for Newtonian fluids that are homogeneous within the scattering volume for flow-SANS measurements, and strong enough to deform complex fluid microstructures. After experimentally confirming the predicted flow behavior for a Newtonian fluid (glycerol), we then investigate the ability to produce nearly 2D flows with controllably variable flow types and deformation rates over a range of fluids with non-Newtonian rheological behavior using particle tracking velocimetry (PTV) measurements. Flow-SANS measurements are then demonstrated for arbitrary deformation types, and applied to measuring flow-induced changes in the microstructure of a dispersion of rod-like cellulose nanocrystals (CNCs) in deuterated water/glycerol (ratio 90:10).

## Device Simulation and Parameterization

As indicated in the preceding section, we believe that the FFoRM geometry based upon the original design of Lee *et al*. has the best chance of satisfying the constraints for simple implementation and interpretation of flow-SANS measurements: namely a spectrum of homogeneous, 2D flows with sufficient flow strength and enough accumulated strain for the material to reach a steady state configuration.

**Figure 3:**
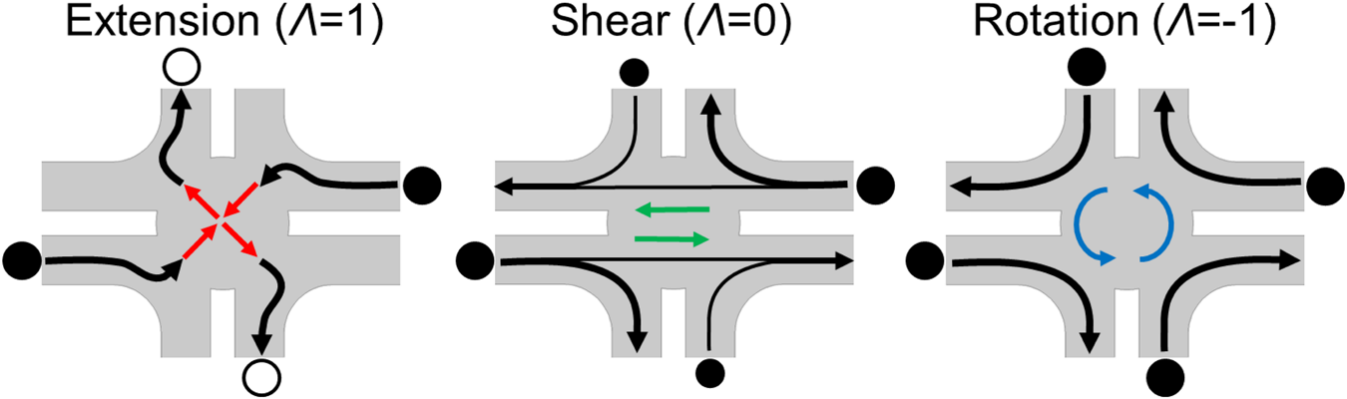
Scheme for generating extensional, shearing, and rotational flows in the fluidic four-roll mill. The black circles indicate the direction and magnitude at which fluid is supplied to (filled circles) or withdrawn from (open circles) the device. The colored arrows represent the nominally generated flow type and black arrows indicate the direction and magnitude of flow rates in the inlet/outlet channels.

To accommodate the flows of non-Newtonian fluids, we modified the Lee *et al*. design by removing the sharp protrusions from the channel dividers at the inlet to the center region. The elimination of these sharp protrusions and corners suppresses the tendency to produce non-axisymmetric or unstable flows due to the elastic instabilities associated with streamline curvature for viscoelastic fluids^26,27^. Furthermore, it also enables fabrication protocols that are less prone to machining errors that affect flow stability, while not compromising the device’s ability to generate all flow types. These modifications allow for a simpler device geometry that can be parameterized by the scheme illustrated in Fig. 1. By specifying the flow rates in specific channels, the FFoRM can be controlled to produce arbitrary 2D flows for Newtonian fluids. This is schematically illustrated in Fig. 3 for the generation of extensional, simple shear, and rotational flows. Approximately, the average of the flow rates in the inlets and outlets (*Q*_2_ + *Q*_1_)/2 determines the magnitude of the velocity gradient, while the ratio of these two flow rates (*Q*_1_/*Q*_2_) determines the flow type.

Generally, devices with larger dimensions can generate flows that are spatially uniform over a larger area. However, they are limited in their capacity to generate flows at high strain-rates before fluid inertia influences the flow, as indicated by the Reynolds number (*Re*) which characterizes the relative magnitude of inertial contributions to the fluid momentum relative to the viscous stress

4 $$Re=\frac{\rho UW}{\eta }=\frac{\rho \frac{{Q}_{2}}{HW}W}{\eta }=\frac{\rho {Q}_{2}}{\eta H}.$$

Here, *ρ* is the fluid density, *η* its viscosity, and *H* is the inlet channel height. With respect to fluid flow, high aspect ratio (*H*/*W*) devices are ideal to eliminate 3D flow effects (*e.g*. large gradients through the thickness of the device from the upper and lower boundaries) when the possibility for creating slip surfaces (e.g. through a lubricating fluid layer or chemically/physically modified surfaces) is infeasible^28^. In SANS measurements, however, lubricating fluids or surface modification can lead to background scattering that interfere with measurements while thick samples lead to multiple scattering and low beam transmission. For this study, we have fixed the thickness of the device *H* = 3 mm as a compromise to mitigate 3D flow effects while still minimizing multiple scattering. For the current setup with achievable pump flow rates ranging from ~10^−4^ μL/hr to 220 mL/min (Harvard Apparatus PHD 2000), *Re* can theoretically vary from 10^−9^ to 1 for a fluid with *ρ* = 1.0 g/mL and *η* = 1.0 Pa s. However, the pump’s torque limitations (~400 kPa) provide an additional constraint on the maximum flow rate that is dependent on the fluid viscosity. For the current tubing size and length, we estimate this maximum to be ${Q}_{max} \sim \frac{20\,\mathrm{mL}/\,\min }{\eta \,[Pa\,s]}$. For the purpose of flow simulations, we only consider the impact of *Re* as this provides a physical constraint on the device operation. Additionally, the neutron beam spot is collimated to a 1 mm diameter circle that ensures adequate signal from the SANS measurement. In the final device configuration described later, we will show that the 3 mm device thickness ensures that the magnitude of in-plane gradients represents a large percentage of the total magnitude of gradients in the beam region of the device, thereby justifying the assumption of nearly 2D flow in the device.

2D CFD simulations were used to determine the impact of the geometric parameters shown in Fig. 1 on the deformations (rate and type) achievable and the uniformity of the flow in the center plane of the device near the stagnation point. Using *COMSOL Multiphysics* software, the Navier-Stokes and continuity equations (for a Newtonian fluid) were solved numerically for the velocity and stress fields in the device. In order to ensure the device design is sufficient for the previously stated objectives, we define our objective to minimize flow non-uniformity (which we define as the standard deviation of *Λ* within the beam diameter at the center-plane of the device) and maximize the deformation rate (defined as the average deformation rate in the center plane beam region) for a given value of *Re* at the flow types of interest. For the Newtonian fluid, we found that the maximum *Re* achievable before significant flow modification due to inertia was 0.2, Therefore, the impact of geometric changes were investigated at constant *Re* = 0.1 so that the deformation rates calculated are near the maximum achievable in the center plane of the geometry before inertial flow modification. In this work, we choose to limit our focus to the uniformity of the flow type rather than the uniformity of the deformation rate. Non-uniformities in flow type and deformation rate varied similarly with geometric changes, but with higher magnitude for variations in the flow type. With regard to non-uniformity in the flow type, the objective is to ensure that the device produces flows with a maximum standard deviation of center-plane flow type of *σ*_Λ_ ≤ 0.1 for all *Q*_1_*/Q*_2_. Note that this is a lower bound estimate of the uniformity of deformation experienced by the fluid in the probing beam region, since as will be shown later the region of uniform flow may extend beyond the beam region in certain directions.

More detail on the device optimization can be found in the Supplementary Material Section B. After systematically varying all geometric parameters (*C*, *W*, *D*, and *R*) in increments of 0.5 mm, we found that the geometry that generates the highest deformation rates while maintaining *σ*_Λ_ ≤ 0.1 for all *Q*_1_*/Q*_2_ was a geometry with *C* = 5 mm *W* = 2 mm, *D* = 1 mm, and *R* = 1 mm. For the Newtonian fluid glycerol at 20 °C (with *ρ = *1.261 g/mL and *η* = 1.412 Pa s), this device geometry generates maximum deformation rates (when *Re* = 0.1) of $\dot{\Gamma }\ge 10\,{s}^{-1}$ for all flow types. For water at 20 °C (*ρ = *1.00 g/mL and *η* = 10^−3^ Pa s), the device generates maximum deformation rates $\dot{\Gamma }\ge 0.01\,{{\rm{s}}}^{-1}$ for all flow types.

For a circular region of homogeneous, linear flow, the average amount of strain accumulated at material points within the region can be numerically estimated assuming there is no strain prior to entering this region. The value varies depending on the flow type, ranging from 1.1 for purely extensional flows to 0.53 for nearly shear flows (*Λ* = 0.01), but is independent of the flow rate. Furthermore, the fraction of the beam that has accumulated a strain of 1 or more varies from 0.55 for an extensional flow to 0.83 for a near shear flow (see Supplementary Materials Section C). The strain accumulated in a flow with closed streamlines (*Λ* ≤ 0) is infinite. These estimations, based only on the flow within the beam, are conservative in the sense that any extension of the homogeneous flow outside the beam is neglected. The Lagrangian convection of microstructures from these regions into the beam region will contribute to the accumulated strain, and hence to greater uniformity of microstructural configurations within the beam compared to an estimate that assumes no strain is accumulated prior to entering the beam region.

In principle, a similar geometry design process could be used to optimize the device for other criteria (e.g. higher deformation rates or accumulated strain) or for other model fluids by utilizing different constitutive models in the generation of flow fields with CFD. However, in what follows we evaluate the device designed above for Newtonian fluids to assess its capabilities for generating stable flows for several types of complex fluids.

## Experimental Evaluation of the Flow Fields For Newtonian and Non-Newtonian Fluids

The FFoRM device developed above is based on the ability of the flow device to generate homogeneous flows of Newtonian fluids within at least the scattering region centered about the central stagnation point. In this section, we use flow visualization experiments to characterize the flows that are actually generated in the device (Fig. 2b,c) for Newtonian and four ubiquitous classes of non-Newtonian fluids (shear thinning, yield stress, elastic (Boger), and viscoelastic). This investigation is intended to address utility of the current FFoRM geometry for several complex fluids, identifying those fluids for which the current design is satisfactory, and the challenges that are encountered for other types of fluids.

### Newtonian fluid behavior

We begin with measurements for a Newtonian fluid. It is expected that the measured behavior will be accurately predicted by the numerical simulations from the design process, but comparison of measured and predicted data will provide some indication of our ability to experimentally determine the velocity gradient using particle tracking velocimetry (PTV). In addition, we utilize 3D simulations to evaluate any out-of-plane flows or out-of-plane velocity gradients that may develop for a Newtonian fluid as a result of the device’s finite thickness (these will be assessed by experiments on the shear thinning fluid to follow). These 3D simulations were carried out using COMSOL Multiphysics software with similar specifications as the 2D simulations, but with added no slip boundary conditions on the upper and lower walls. The PTV measurements were made by seeding the test fluid with 10 μm hollow glass spheres in dilute (300 ppm) concentrations and capturing their in-plane trajectories in the center plane and at the stagnation point of the device. Using well developed algorithms for tracking tracer particles between frames and determining their velocity fields, we determine the spatial in-plane velocity fields and, subsequently, the in-plane velocity gradient fields by numerical differentiation with a locally weighted least squares regression^29,30^.

**Figure 4:**
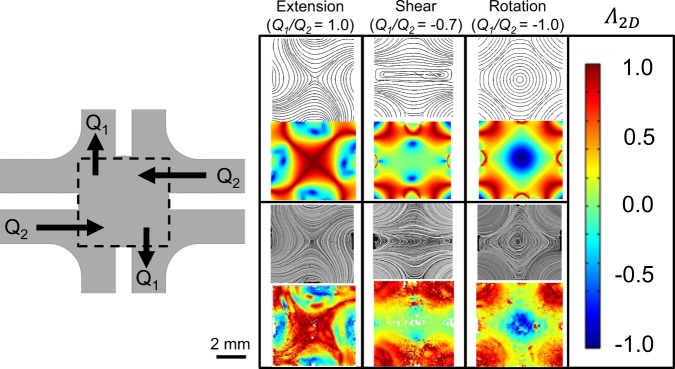
Streamlines (upper) and local 2D flow type parameter (*Λ*_2*D*_, lower) comparisons between simulated (top) and experimentally determined (bottom) flow of glycerol (Newtonian fluid) in the center plane of the FFoRM geometry. All tests correspond to conditions with constant *Q*_*2*_ = 0.5 mL/min (*Re* = 0.0025) and the values of *Q*_1_*/Q*_2_ indicated. Experimental conditions correspond to 〈$\dot{\Gamma }$〉 ~0.5 s^−1^ for extension, ~0.25 s^−1^ for shear, and ~0.2 s^−1^ for rotation. The device geometry is included on the left with the region of interest for PTV experiments outlined for reference.

Streamlines at the center-plane of the flow device as predicted by simulation are compared to experimental streakline images generated by superposing PTV images from a single captured video. These results are included in Fig. 4 for three representative flows (extension, shear, and rotation) at constant *Q*_2_ = 0.5 mL/min (*Re* = 0.0035). Likewise, simulated and experimental 2D flow type parameters (*Λ*_2*D*_) at the center-plane are spatially evaluated from the 2D velocity gradient tensor and are used as a scalar comparison of the full velocity gradient tensor. White regions in the experimental indicate area where no particles were tracked, and noise in the experimental data can be attributed to tracking errors^29^ as well as small (~4%) variations in fluid velocity through the finite focal depth of measurement (~0.3 mm). Experiments show no particles moving in or out of the depth of field, indicating that, near the center plane, fully 3D flows are minimal. We note that the location of the stagnation point can shift from the center when operating at conditions near shear flow (*Λ*_2*D*_ ~ 0). We attribute this shift to small variations in ambient pressure, which can drive a shift along the center line of the device (left to right in Fig. 4) where fluid velocity is near zero. Similar difficulties were reported in Lee *et al*.^21^ and we further note that this shift in stagnation point does not noticeably change the flow type generated at the center of the device nor the long residence time in the beam region.

**Figure 5:**
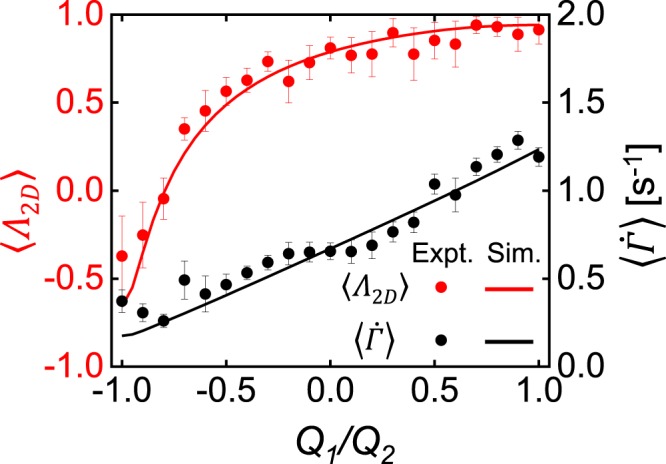
Quantitative comparison of flows of a Newtonian fluid in the FFoRM geometry determined by experiment (points) and simulation (lines). Variations in the volume averaged 2D flow type parameter (*Λ*_2*D*_, red) and magnitude of velocity gradient tensor (〈$\dot{\Gamma }$〉, black) are plotted as a function of the operating parameter (*Q*_1_/*Q*_2_) for constant *Q*_2_ = 1.0 mL/min (*Re* = 0.005). Error bars in the experimental measurements indicate the standard deviations in the beam region of the measured average value.

To quantitatively compare the flows of Newtonian fluids experimentally generated in the FFoRM with simulations, we calculate volume-averaged values of the flow type parameter and magnitude of the velocity gradient tensor for a region near the stagnation point in the center of the device. In the experiments, we choose a 1 mm diameter circular averaging region to match the region of uniform flow that the device was designed to achieve. In the simulations, the averaging volume is a 1 mm diameter, and 0.3 mm height cylindrical region around the nominal stagnation point in the center plane of the device chosen to match the focal depth of the experimental imaging system. Representative results are shown in Fig. 5 as a function of the ratio of volumetric flow rates *Q*_1_/*Q*_2_ for a constant value of *Q*_2_ = 1.0 mL/min (*Re* = 0.007). As with the spatially mapped flow type parameter, the numerical values of *Λ*_2*D*_ and $\dot{\Gamma }$ from simulations and experiments of a Newtonian fluid agree quantitatively, as we should expect.

### Non-Newtonian flow behavior

Four representative complex fluids were formulated and rheological measurements under shear in a cone-plate rheometer were used to determine the steady state shear viscosity for a range of applied shear rates. Details of these formulations and measurements are included in the Methods section. Summarizing, the fluids were formulated with relaxation time scales that insured nonlinear rheological response for shear rates/ velocity gradients in the range less than 20 s^−1^ that can be realized in the flow device with viscosities in the range 0.1–5 Pa s.

#### Inelastic shear-thinning fluid

**Figure 6:**
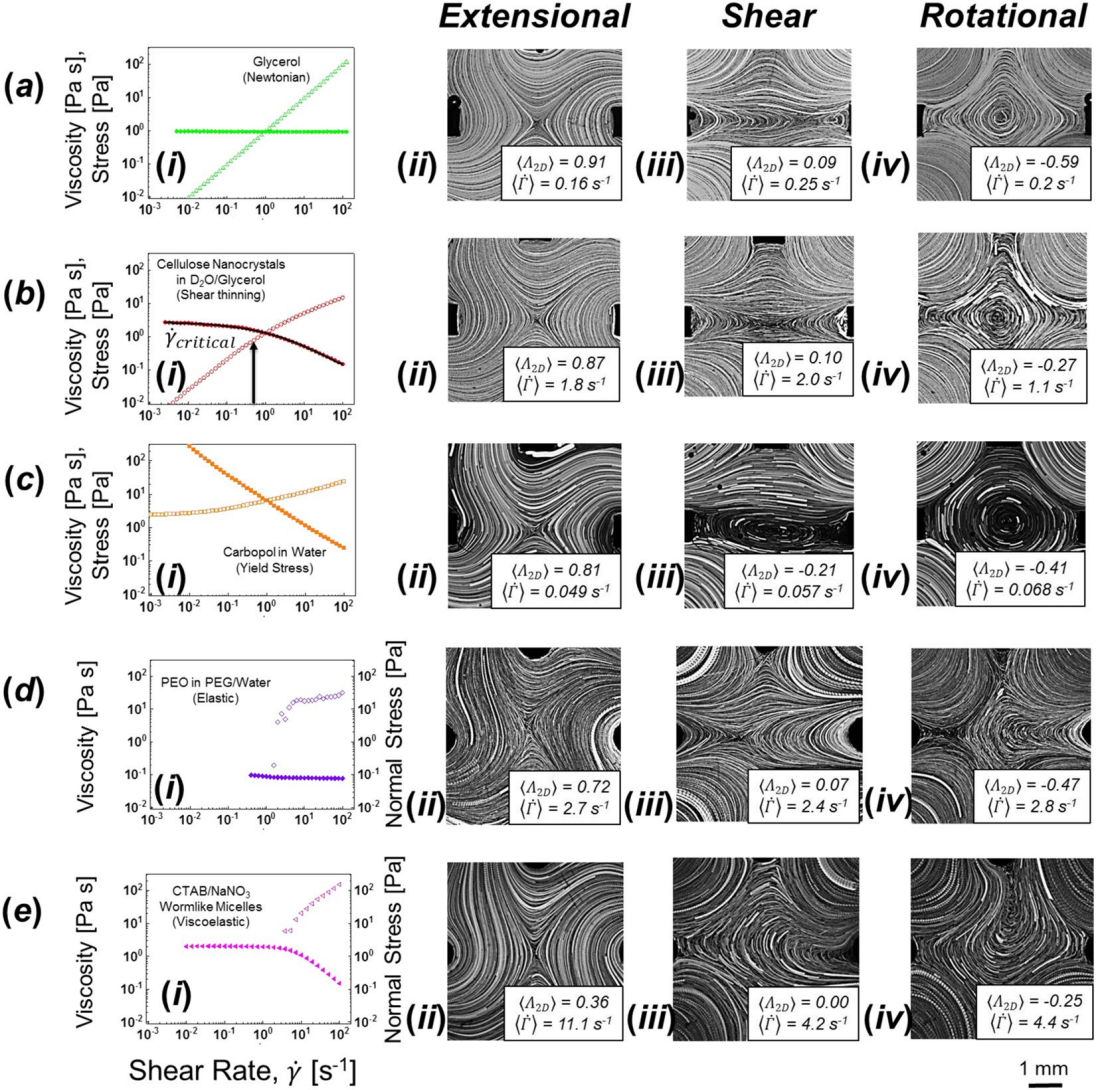
Steady shear rheology (left, *i*) and streaklines for conditions that generate extensional, shear and rotational flows for a Newtonian fluid (from left to right, *ii* to *iv*) for (**a**) a Newtonian fluid and several fluids exhibiting non-Newtonian shear rheological responses: (**b**) shear thinning, (**c**) yield stress, (**d**) elastic, (**e**) viscoelastic. Operating conditions are set to those that generate extensional (*Q*_1_/*Q*_2_ = 1), shear (*Q*_1_/*Q*_2_ = −0.7), and rotational (*Q*_1_/*Q*_2_ = −1) flows of the Newtonian fluid. Steady shear rheology plots include viscosity (filled shapes) and shear stress (open shapes) as a function of the shear rate for all fluids except the viscoelastic fluid for which the normal stress is included instead of shear stress. The solid line in *(bi)* is a best fit of the viscosity data to the Carreau model.

An aqueous dispersion of rigid, rod-like cellulose nanocrystals (CNCs) was used as a characteristic shear thinning fluid with minimal elasticity. CNCs derived from wood, such as those used in this study, have been found to be parallelepipeds with length, width, and height in the range of 100–200, 10–20 nm, and 2–5 nm respectively^31–33^. Such non-spherical particle dispersions exhibit flow-induced particle alignment in an imposed straining flow, which modifies their contribution to the fluid’s viscosity (thinning in a shear flow and thickening in an extensional flow)^33,34^. Rheological measurements were used to identify 5 wt% CNC in 85.5 wt% D_2_O and 9.5 wt% glycerol as a model inelastic shear thinning fluid. The CNC concentration was chosen as the highest concentration before the emergence of nematic domains (indicated by the presence of birefringence at rest). The choice of solvent is a compromise between increased fluid-particle contrast in the SANS measurements and increased suspending medium viscosity (reducing the rotational diffusivity and thereby reducing the strain rates required for flow-induced orientation). The steady state shear response of the CNC dispersion is typical of a shear thinning fluid, and is characterized by a pseudo-Newtonian plateau in the viscosity at low shear rates with a zero-shear viscosity *η*_0_ = 2.50 Pa s, followed by the onset of shear thinning at a shear rate of 0.5 s^−1^ (Fig. 6bi).

For such a semi-dilute suspension of orientable particles, the degree to which particles align is governed by the rotational Peclét number,

5 $$P{e}_{r}=\frac{|{\bf{E}}|}{{D}_{r}},$$

where *D*_*r*_ is the long-time collective rotational diffusivity of a particle in the dispersion, which can be related to that for a single, dilute rod^35^. Note that |***E***| is used as the characteristic deformation rate. Consequently, the alignment of the dispersion is dependent only on the rate of strain, and not the flow type. With these definitions, the onset of shear thinning occurs for *Pe*_*r*_ = *O*(1). Thus, the shear rate corresponding to the onset of shear thinning ($\dot{\gamma }$ = 0.5 s^−1^) is used as an estimate the effective rotational diffusivity for this non-dilute system, *i.e*., *D*_*r*_ = 0.5 s^−1^.

Representative streakline images of flows, along with magnitudes of the experimentally observed flow type parameter and velocity gradient tensor, are reported in Fig. 6b for a nominal applied Peclét number of 4, where the shear thinning exponent is approximately n = 0.3. Under these conditions, we find qualitatively similar flow patterns to those observed for the Newtonian fluid (shown in Fig. 6a). The largest impact from shear thinning rheology is in the rotational flow case, where the deformation rate generated is reduced by half from the nominal (Newtonian) value. We hypothesize that this effect is due to thinning of the fluid in the entry channels which reduces the shear stress acting on (and tending to rotate) the fluid in the central region of the geometry. Other than this difference, the flows generated match the flows of a Newtonian fluid, suggesting that shear thinning alone has little effect on the ability to achieve arbitrarily variable flow types in the FFoRM at least for this degree of shear-thinning.

#### Yield stress fluid

A dilute, aqueous solution of Carbopol 934 was formulated for use as a model yield stress fluid. Aqueous dispersions of neutralized Carbopol 934 display yielding behavior^36^. From rheological measurements (Fig. 6ci), the value of the yield stress is quantified by the plateau in stress at low shear rates. The concentration of Carbopol 934 (0.2 wt%) was chosen so that the fluid exhibits a moderate yield stress (2.4 Pa) upon neutralization with 25 wt% KOH. It should be noted that the fluid is very strongly shear-thinning beyond the yield stress.

Streakline images of the carbopol solution in the FFoRM at low nominal deformation rates (${\dot{\Gamma }}_{nom}$ ~ 0.05 s^−1^) are included in Fig. 6c. Qualitative differences from Newtonian behavior are noticeable in the streaklines for *Q*_1_/*Q*_2_ values that produce shear and rotational flows for Newtonian fluids. Operation under nominal shear conditions (*Λ*_2*D*_ ~ 0) produces a flow that is more rotational (*Λ*_2*D*_ < 0). Streaklines for the rotational flow condition display a larger region of closed streaklines than for the Newtonian case. We attribute these differences in the flow fields to a more plug-like flow in the entry channels due to the strong shear thinning of the fluid near the confining walls. We find that flows can be tuned to produce the full range of flow types from extension to rotation, albeit with different operating conditions compared to a Newtonian fluid for the desired flow type that must be determined through velocimetry experiments (or, in principle, *via* simulations for a corresponding rheological constitutive model).

#### Purely elastic fluid

A polyethylene oxide (PEO) based Boger (purely elastic) fluid was formulated to study the impact of elasticity on flows in the FFoRM. Boger fluids are a class of elastic fluids that have a constant viscosity in shear flow over a range of deformation rates, but significant normal stress differences. Under steady flow conditions in a general flow, the strain rate relative to the fluid’s elastic relaxation time (λ) is given by the Weissenberg number

6 $$Wi=\lambda |{\bf{E}}|$$

We formulated a water-based PEO Boger fluid such that it has a nearly constant shear viscosity (*η* = 0.08 Pa s) up to 100 s^−1^ and λ ~ 0.16 s (Fig. 6di)^37^.

Streakline images of the elastic fluid flows for *Wi* = 2 are included in Fig. 6d and show significant flow modification from the nominal (Newtonian fluid) behavior. We find that, for flows nominally dominated by extensional deformation, this flow modification tends to generate flows with flow types closer to shear, presumably as a way to minimize extensional stresses. For flows nominally dominated by shear and/or rotation, flow modification begins upstream of the stagnation point as the fluid rounds the entry corner into the central region, which then significantly influences the behavior near the stagnation point. We note, however, that for nominally straining flows (*Λ*_2*D*_ > 0), the flow typically remains stable, i.e., it achieves a steady state that is invariant in time over the periods of measurement. By contrast, in the shear and rotational cases (*Λ*_2*D*_ < 0), the flows also tend to be unsteady, as indicated by streaklines that cross one another.

**Figure 7:**
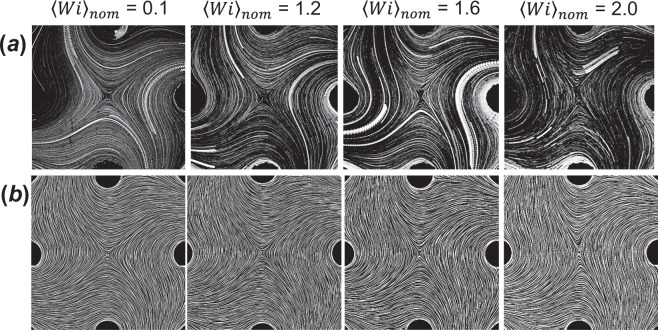
Streakline and streamline images of flows in the FFoRM geometry for (**a**) PTV experiments with an elastic Boger fluid and (**b**) CFD simulations of an Oldroyd-B fluid. Device operation conditions are for nominally extensional flows (*Q*_1_/*Q*_2_ = 1) with nominal center plane deformation rates, *Wi*_*nom*_, indicated above images.

To further characterize the development of flow modification for the steady, nominally straining cases, we examine the development of flow modification with increasing nominal *Wi* for the purely elastic Boger fluid with *Λ*_2*D*_ ~ 1. Figure 7a includes streakline images from experiments at several values of the corresponding nominal *Wi*. Below *Wi* = 1, the flow is unmodified and matches that of a Newtonian fluid. As the nominal deformation rate is increased beyond *Wi* = 1, the streaklines modify from their unperturbed hyperbolic trajectories, generating flows with increasingly more vorticity. Simulations were carried out in the 2D FFoRM geometry for an Oldroyd-B (purely elastic) fluid with parameters chosen to match the formulated Boger fluid (*η*_*solvent*_ = 0.057 Pa s, *η*_*polymer*_ = 0.043 Pa s, and λ = 0.158 s) using the *OpenFOAM* software package^38,39^. Figure. 7b includes the streamline images for the flow fields at the corresponding experimental conditions. We find that the Oldroyd-B model captures the flow modification observed experimentally. These results are consistent with earlier work, where it was reported that stretching type deformations of elastic fluids tend to be minimized due to large extensional stresses^40–42^. We thus conclude that the inability to generate extension dominated flows of elastic fluids when *Wi* > 1 is a physical limitation due to the fluid’s rheology that remains as a significant challenge for the FFoRM type devices, or any device in which the fluid experiences an abrupt change in cross-sectional geometry.

#### Viscoelastic Fluids

A solution of CTAB/NaNO_3_ wormlike micelles (WLMs) was formulated to investigate the impact of viscoelasticity (elasticity with shear thinning) on flows in the FFoRM^43^. Wormlike micelles are long, flexible chains formed by surfactants. Due to the noncovalent nature of the interactions that give rise to chains, in contrast to polymer solutions, wormlike micelles exhibit dynamic scission and reformation^44^. Despite this difference, the surfactant chains can entangle to form a viscoelastic network with a mechanical response that is similar to entangled polymer solutions. We formulated a 100 mM CTAB/300 mM NaNO_3_ solution in water that shows a viscoelastic response (λ ~ 0.3 s) (Fig. 6ei) at ambient temperature^43^.

The observed flow behavior is shown for *Wi = *2 in Fig. 6e. Qualitatively, the flows are similar to those for the elastic fluid. However, the tendency to produce a weaker (more rotational) flow is enhanced under conditions that would produce an extensional flow with *Wi* = 2.0 for a Newtonian fluid, with *Λ*_2D_ being only 0.36. Furthermore and similar to the purely elastic fluid, the shear and rotational flow fields are significantly corrupted and also appear to be unsteady.

### FFoRM Operating Limits

**Figure 8:**
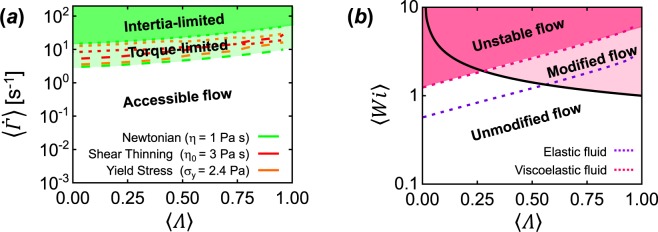
Operating diagrams indicating accessible flows in the FFoRM. (**a**) Operational limits for average flow type (*Λ*) and magnitude of velocity gradient tensor (〈$\dot{\Gamma }$〉) for inelastic fluids tested in the FFoRM under strong flow conditions (*Λ* > 0). Practical limitations due to inertia (dotted lines) or the pump torque (dashed lines) define an upper limit on 〈$\dot{\Gamma }$〉 for a given *Λ* in the current setup for the fluids indicated (colored lines). (**b**) Limits for *Λ* and 〈*Wi*〉 for elastic and viscoelastic fluids tested in the FFoRM. Practical limitations due to inertia (dotted lines) or physical limitations due to the fluid elasticity (solid black line) define the upper limit of 〈*Wi*〉 for a given *Λ*, defining regions of modified (steady) and unstable (unsteady) flow.

Given the flows produced for various rheological responses, it is important to summarize the limits of operation of the FFoRM device with respect to both maximum deformation rate and flow type placed by the fluid (including inertia or elasticity) as well as by equipment limitations, such as torque limitations of the pumps used to drive the flow (Fig. 8). Inertial limitations, which we find occur when *Re* > 0.2, can be overcome for a particular fluid by scaling down the size of the device. Torque limitations can be overcome by using pumps with a higher torque limit. These limits place upper bounds on the accessible space of $\dot{\Gamma }$ and *Λ*_2*D*_ achievable in the FFoRM for any fluid (Fig. 8a). Furthermore, fluid elasticity leads to flows that are significantly modified away from the nominally applied flow set by *Q*_1_/*Q*_2_ (when *Wi* > 1 and *Re* $\ll$ 1), or are unsteady in time (when *Wi* > 1 and *Re* > 1). These limits place further bounds on the accessible space of 〈*Wi*〉 and *Λ*_2*D*_achievable in the FFoRM for fluids with appreciable elasticity (Fig. 8b). Efforts to overcome the inability to generate extension dominated flows of elastic and viscoelastic fluids will be the subject of future work, based upon different designs of the flow geometry.

We conclude that the FFoRM geometry in its current configuration has significant limitations when used with elastic or viscoelastic fluids. It can provide the full range of flow fields for inelastic shear thinning fluids and for yield stress fluids in the rheologically interesting range of deformation rates. This does not mean that it cannot be usefully applied for elastic and viscoelastic fluids, but it will be necessary to have detailed knowledge of the flow fields in order to interpret SANS-based data on the configuration of the microstructure for such fluids. Fortunately, for purely elastic fluids, the Oldroyd B constitutive model can be used to obtain detailed predictions of the flow. For viscoelastic fluids, the situation is less clear, but likely one would have to experimentally characterize the flow in order to interpret such measurements.

## Flow-SANS Study of a CNC Dispersion Using the FFoRM

Following these initial investigations into the flow behavior of non-Newtonian fluids in the FFoRM, we seek to validate that the proposed FFoRM-SANS workflow (Fig. 2) yields interpretable results for a fluid whose microstructural response under flow is predictable. To accomplish this validation, we investigated the CNC dispersion that was previously described as a model inelastic shear thinning fluid, since the direction of orientation of nonspherical particles at low *Pe*_*r*_ may be predicted even without the benefit of a rheological model.

In this section, we discuss the full spectrum of experiments required for FFoRM-SANS to probe the orientation of the CNCs near the stagnation point in so-called strong flows (*Λ*_2*D*_ > 0), where we expect measurable microstructure orientation due to a combination of hydrodynamic and interparticle interactions^22^. In particular, we first measure the velocity and velocity gradient fields generated in the device using PTV for quantitative comparison to CFD simulations of a model inelastic shear thinning fluid (Fig. 2a–c). The measured microstructural response in the dispersion determined by SANS is then compared to asymptotic results for the orientation distribution of dilute rods in steady, homogeneous 2D flow to validate that the FFoRM achieves a measurable steady-state microstructural response (Fig. 2d).

### CNC dispersion flow visualization

In order to validate the ability to generate nearly homogeneous 2D flows of the CNC dispersion in the FFoRM, we now investigate in greater detail the fluid’s flow uniformity and stability in the device. If uniform flows are generated in the probing beam region of the device, this provides confidence that FFoRM-SANS measurements will probe the CNC structure under the desired, uniform flow conditions, provided that the strain accumulated is sufficient for the dispersion to reach steady state. PTV was performed in a similar manner to the experiments for the Newtonian fluid, and velocity gradients were calculated for fluid flows near the mid-plane of the device. Furthermore, using this data, we seek to validate the use of a simple generalized Newtonian fluid (GNF) model – in this case, the Carreau model fit to the shear rheology data in Fig. 6bi – for numerical (CFD) simulations of the flow. The model, including best-fit values of the parameters, is reported in the method section. The CFD simulations were performed in *COMSOL Multiphysics* with the same 2D and 3D FFoRM geometries and corresponding boundary conditions. If successful, this validation indicates that CFD simulations can be used to predict operating conditions corresponding to a particular generated flow condition, circumventing the need for flow measurements at all possible flow conditions.

**Figure 9:**
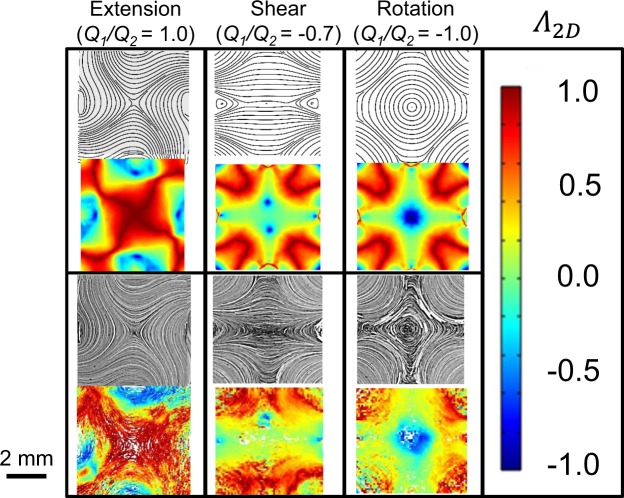
Streamlines (upper) and local flow type parameter (*Λ*_2*D*_, lower) comparisons between simulated (top) and experimentally determined (bottom) flow of a 5 wt% CNC dispersion (shear thinning fluid) at the center plane of the FFoRM geometry. All test conditions correspond to *Q*_*2*_ = 1.0 mL/min (*Re* = 0.005) and the values of *Q*_1_*/Q*_2_ indicated. Experimental conditions correspond to 〈*Pe*_*r*_〉 ~5 for extension, ~3 for shear, and ~1 for rotation in the center plane of the device.

**Figure 10:**
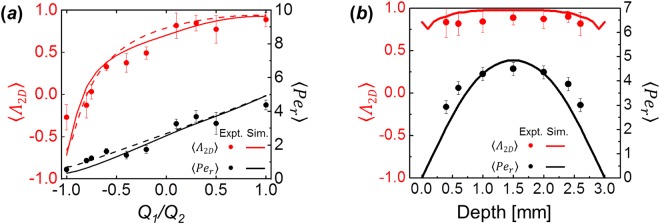
Experimental (points) and simulated (lines) flow mapping for a shear thinning fluid in the FFoRM geometry. Simulation results included are for a GNF fluid with viscosity described by the Carreau model (solid lines). Results for a Newtonian fluid are included for reference (dashed lines). The volume averaged 2D flow type parameter (*Λ*_2*D*_, red) and rotational Peclét number (〈*Pe*_*r*_〉, black) are included as a function of (**a**) the operating parameter (*Q*_1_/*Q*_2_) at the center plane of the geometry for constant *Q*_2_ = 1.0 mL/min (*Re* = 0.005) and (**b**) the depth through the device for constant *Q*_1_* = Q*_2_ = 1.0 mL/min (*Re* = 0.005). Error bars in the experimental measurements are the standard deviations of the measured average value in the beam region.

Representative results from the 2D CFD simulations and PTV experiments (Fig. 9) demonstrate that the FFoRM can generate extensional, shear, and rotational flows (and arbitrary combinations thereof) for the CNC dispersion well into the shear thinning regime. When evaluating the differences between the flows of a Newtonian fluid (Fig. 4) and the shear thinning CNC dispersion (Fig. 9), we find only minor differences in device operation (Fig. 10, solid and dotted lines). Notably, the shear thinning of the suspension decreases the maximum deformation rates achievable before *Re* = 0.2 by approximately half. As with the Newtonian fluid, we find excellent agreement in the flow type mapping between the Carreau fluid simulations and experiments with the CNC dispersion. This finding was not necessarily expected given that our choice of constitutive model does not show an extension thickening response that one would expect from microstructural models of rod-like suspension microstructure^45^. We note that this agreement is likely due to only moderate increases in the extensional viscosity as predicted for semi-dilute, hydrodynamically interacting rods of the dimensions and concentrations that we are investigating (~2 times that of a Carreau fluid for *Pe*_*r*_ $\ll$ 1)^46^.

As we did for the flows of the Newtonian fluid, we map the flow fields generated for the CNC dispersion under nonlinear flow conditions (*Pe*_*r*_  > 1) in a 1 mm circular area near the stagnation point for quantitative comparison of generated velocity gradients in 3D CFD simulation and experiment (Fig. 10a). We find good agreement in both the generated deformation rate magnitude and flow type. As noted already, the addition of shear thinning only slightly modifies the operation of the FFoRM. Additionally, we measure the variation of velocity gradient through the thickness of the device under extensional flow conditions (*Pe*_*r*_ = 3.5, *Λ*_2*D*_ = 0.93) to assess the 3D variation in the out-of-plane direction (Fig. 10b). We find that the simulations capture the variation in the magnitude of the strain rates through the thickness of the device. We note that the simulations predict a more parabolic variation in the strain rate through the device than the experiments, which indicate a plateau of nearly constant strain rate at the center of the device. We attribute these differences to particles being tracked from outside the focal plane of the measurement which would lead to a smoothing of the parabolic profile. From these measurements, we consider the simulations to provide a good representation of the flows generated for the CNC dispersion. Furthermore, the experiments indicate that significant gradients along the device thickness (coincident with the SANS beam path) only occur in a region within 10% of the confining walls of the device. As such, the flow can be approximated as an effective 2D flow, which significantly simplifies the interpretation of the SANS data to follow. Moreover, with the fluid flow mapping of the CNC dispersion in the FFoRM geometry as well as a validation of the CFD simulations, we gain the ability of imposing arbitrary, uniform flows of the nanoparticle dispersion in the beam region of the device.

### FFoRM-SANS measurements and data analysis

We turn to validate the final step in the FFoRM-SANS workflow (Fig. 2d), measuring the microstructural response of the CNC dispersion using FFoRM-SANS. Validation of the FFoRM-SANS technique requires that we observe nanoparticle orientation under flow conditions consistent with previous studies of similar dispersions, including more recent experimental studies on CNC dispersions in shear flows^33,47^, and models of colloidal nanoparticle orientation under steady flows^45^. In this validation study, we seek to measure the absolute direction and strength (how probable the rods are to exist in the aligned conformation) of orientation as we vary the flow type and strain rate.

FFoRM-SANS measurements were performed to determine how the microstructure of the CNC dispersion responds under flow at moderate strain rates relative to their effective rotational diffusivity (as indicated by *Pe*_*r*_, equation (5)). Large changes in the orientation distribution of rod-like dispersions are expected in extension dominated (“strong”) flows (*Λ*_2*D*_ > 0) in the nonlinear regime (*Pe*_*r*_ > 1). To achieve these deformations in the FFoRM, we use the previously validated 3D simulations to calculate the operating parameters (*Q*_*2*_ and *Q*_1_*/Q*_2_) that yield a desired average range value of *Pe*_*r*_ and *Λ*_2*D*_ through the beam region of the device. As mentioned previously, these values correspond to the average strain rates achieved in the scattering volume including the regions near the upper and lower walls of the device which will generate lower *Pe*_*r*_ (<20% of the total scattering volume experiences less than half of the strain rate generated in the center plane, see Supplementary Materials Fig. S5), albeit with uniform *Λ*_*2D*_. In this study, we performed SANS measurements where *Λ*_2*D*_ was varied from 0 to 1 and *Pe*_*r*_ was varied from 0.02 to 50. Measurement of shear dominated flows at high *Pe*_*r*_ was limited by inertial effects due to the higher volumetric flow rates required to generate shearing flows compared to extensional flows at similar *Pe*_*r*_.

**Figure 11:**
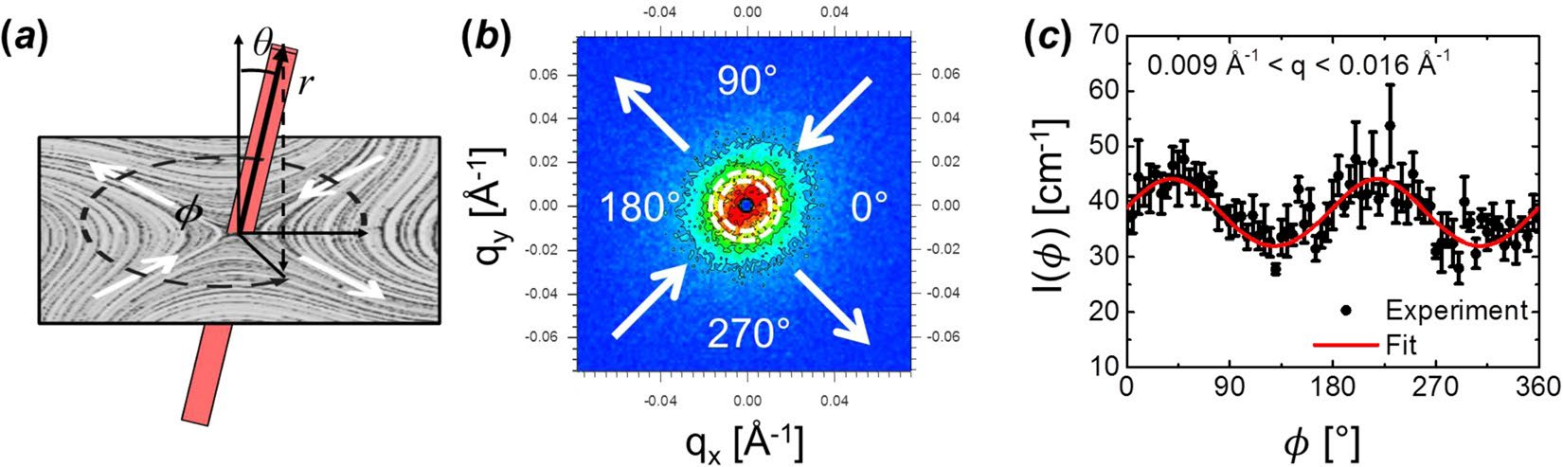
(**a**) Spherical coordinate system defined relative to the velocity-velocity gradient plane for FFoRM-SANS experiments. (**b**) 2D scattering pattern from a FFoRM-SANS experiment with 〈*Λ*_*2D*_〉 = 0.95 and 〈*Pe*_*r*_〉 = 10. Colors correspond to the intensity at (*q*_*x*_*, q*_*y*_) with blue corresponding to low intensity and red corresponding to high intensity while black lines indicate contours of constant intensity. Region included in the annular average (0.009 Å^−1^ < *q* < 0.016 Å^−1^) is outlined in white. Inflow and outflow directions are indicated with white arrows. (**c**) Annular averaged scattering intensity profile (black points) as a function of the azimuthal angle *ϕ* defined relative to the positive *q*_*x*_-direction. The included fit is to the Maier-Saupe function with parameters determined using a nonlinear least squares fitting routine.

For rod-like dispersions such as the CNC solution studied here, the orientational distribution of the particles is reflected in the degree and orientation of anisotropy in the two-dimensional scattering pattern (Fig. 11). In order to quantify the scattering anisotropy, we perform an angle-dependent annular average of the data in the q-range of 0.009–0.016 Å^−1^ to match previous rheo-SANS studies of similar dispersions^33^. The chosen range corresponds to a local maximum in the equilibrium scattering intensity, in which the scattering is dominated by interparticle correlations. As such, the degree of scattering anisotropy in this *q*-range reflects the degree of orientational order of the nanorod dispersion. To quantify this ordering, the angle-dependent scattering intensity was fit to a Maier-Saupe type distribution^48^,

7 $$I(\varphi )={I}_{max}\exp (\alpha (\frac{3\,{\sin }^{2}(2(\varphi -{\varphi }_{0}))}{2}-\frac{1}{2})+\frac{\alpha }{2})$$

using nonlinear least-squares regression where *ϕ* is the azimuthal angle in the *q*_*x*_-*q*_*y*_ (velocity/velocity gradient) plane with *ϕ* = 0 corresponding to the positive-*q*_*x*_ axis and *I*_*max*_, α, and *ϕ*_0_ are adjustable fitting parameters corresponding to the maximum scattering magnitude, amplitude of anisotropy, and the location of the minimum in the scattering intensity respectively, the latter of which represents the average orientation of the microstructure. We note that, as defined, the angle *ϕ*_0_ describes the average orientation relative to the positive-*q*_*x*_ axis in the detector plane, and is defined independently from any local coordinate frame of the flow in the FFoRM device.

A representative scattering analysis is included in Fig. 11, and depicts the azimuthal averaging of 2D data and fitting to extract the orientation angle, *ϕ*_0_. For these anisotropic scattering patterns, we correlate the minimum in the scattering intensity to a maximum in the real space orientation probability distribution function, indicating the most probable particle orientation. Additionally, the alignment factor with respect to annular scattering intensity is calculated numerically using the trapezoidal rule *via*

8 $${A}_{f}=\frac{{\int }_{0}^{2\pi }\,I(\varphi )\,\sin \,(2(\varphi -{\varphi }_{0})d\varphi }{{\int }_{0}^{2\pi }\,I(\varphi )d\varphi }$$

The alignment factor is commonly utilized in flow-SANS experiments to provide a measure of the degree to which the microstructure is aligned along the *ϕ*_0_ direction^48,49^, and ranges from 0 to as high as 1 for isotropic and some perfectly oriented microstructures, respectively.

The modeling of dilute, non-spherical Brownian suspensions subject to arbitrary flows can be accomplished by solving a Fokker-Planck equation describing the orientation probability density function (OPDF) of particle conformations^50^. Microscopically, the effect of increasing strain rate is to preferentially orient particles along the principle strain-rate axis which competes with rotational diffusion due to Brownian motion. While an analytical solution only exists in the case of steady, irrotational flows, asymptotic approximations in the limits of small and large *Pe*_*r*_ can provide insight into the expected behavior of the CNC in the intermediate *Pe*_*r*_ regime^45^. In the limit of low *Pe*_*r*_, the first deviation in the OPDF from isotopic is for the rod-like particles to align along the principal strain-rate axis (see Supplementary Materials Section A). In the limit of large *Pe*_*r*_, particles align along the flow direction in planar flows other than pure shear. From streamline images of the flow, we can determine the absolute orientation of the outflow axis (*ϕ*_*outflow*_). Then, assuming a region of uniform, linear flow within the beam (equation (3)), the absolute direction of the principal strain-rate axis (*ϕ*_*strain*_) is given by

9 $${\varphi }_{strain}={\varphi }_{outflow}-{\tan }^{-1}(\frac{\sqrt{{\Lambda }_{2D}}-1}{\sqrt{{\Lambda }_{2D}}+1}).$$

**Figure 12:**
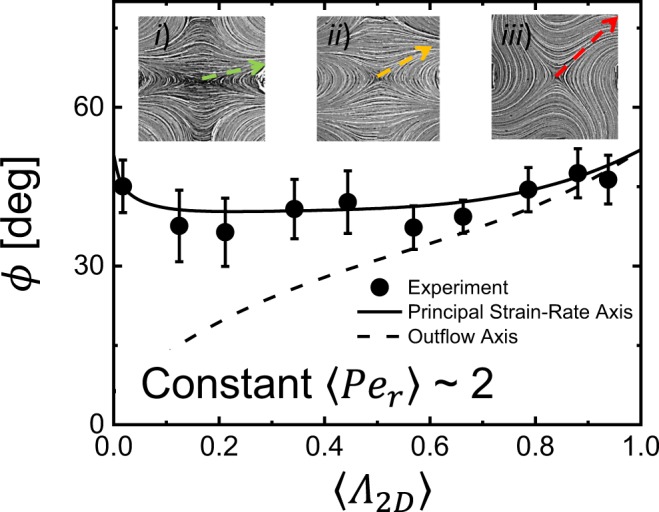
Absolute orientation of CNCs with respect to the *q*_*x*_ direction, *ϕ*, from nearly shear (*Λ*_2*D*_ ~ 0) to nearly extensional (*Λ*_2*D*_ ~ 1) flow for a constant rotational Peclét number *Pe*_*r*_ ~ 2. Lines indicate the absolute direction of the principal strain-rate axis (solid line), *ϕ*_*strain*_, and outflow axis, *ϕ*_*outflow*_ determined from PTV (dotted line). The inset streakline images indicate *ϕ*_*outflow*_ (dotted arrow) for (***i***) *Λ*_2*D*_ = 0.03, (***ii***) *Λ*_2*D*_ = 0.37, and (***iii***) *Λ*_2*D*_ = 0.92. Error bars for *ϕ* correspond to the 68% confidence interval from the nonlinear least squares fit of equation (7).

This enables comparison of the orientation of the principle strain-rate axis *ϕ*_*strain*_ in the scattering (*q*_*x*_ − *q*_*y*_) plane with the average particle orientation *ϕ*_0_ determined from the anisotropic scattering patterns measured in FFoRM-SANS measurements on the CNC dispersion. Again, *ϕ*_*strain*_, *ϕ*_*outflow*_, and *ϕ*_0_ are all defined relative to the *q*_*x*_ direction to allow direct comparison for all applied flow types. Similar to mechanical four-roll mills, the absolute orientation of the principal strain-rate axis in the FFoRM does not vary appreciably with changes in the flow type parameter (Fig. 12), as confirmed through measurements of the outflow axis from simulated streamlines.

Figure 12 shows representative results of this comparison for constant *Pe*_*r*_ ~ 2. For reference, the outflow direction from the stagnation point measured with PTV is also shown. We find that for *Pe*_*r*_ ~ 2, the CNCs align preferentially along the principal strain-rate axis for all flow types from 0 to 1, in agreement with the asymptotic prediction for low *Pe*_*r*_. This agreement between the orientation of CNCs and the principal strain-rate axis means that the current device configuration provides a sufficient amount of accumulated strain to orient the microstructure in the direction dictated by the local deformation field. The implications of this result will be discussed later.

**Figure 13:**
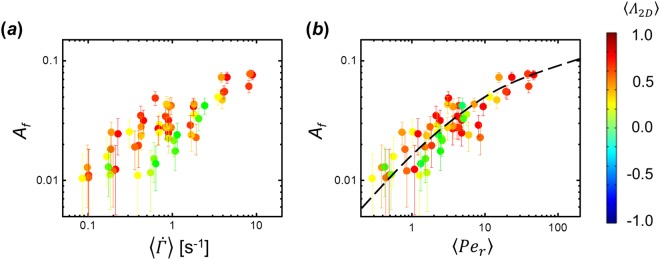
*A*_*f*_ vs. (**a**) average magnitude of velocity gradient tensor ($\dot{\Gamma }$) and (**b**) average rotational Peclét number (*Pe*_*r*_). The color of plotted points indicates the average flow type (*Λ*_2*D*_) where red indicates an extensional flow (*Λ*_2*D*_ = 1) and green indicates a shear flow (*Λ*_2*D*_ = 0). Data for which *A*_*f*_ falls below the lowest resolvable value (*A*_*f*_ ~ 0.01) are suppressed. The black line in (**b**) is a spline fit included as a guide to the eye. Error bars correspond to variability in the SANS pattern over the q-range used in Equation (8).

We further examine the dependence of the degree of alignment, as measured by *A*_*f*_, on various measures of the strength of the flow (Fig. 13). We find that *A*_*f*_ increases monotonically with increasing deformation rate for conditions with similar flow type. However, this trend varies when plotted against 〈$\dot{\Gamma }$〉 (Fig. 13a). If instead the deformation rate is quantified in terms of *Pe*_*r*_ = |**E**|/*D*_*r*_ (*i.e*. the dimensionless rate of strain), the measured *A*_*f*_ values exhibits modest collapse, with a trend that is independent of the flow type. This observation is consistent with the theoretical prediction for dilute, nonspherical particles at low *Pe*_*r*,_ where the orientation distribution function (and therefore the alignment factor) is only a function of the *Pe*_*r*_. This result would be non-obvious in the absence of theoretical predictions, since the collapse of *A*_*f*_ with strain rate is observed despite the larger upstream fluid velocities (proportional to *Q*_*2*_) required to generate flows with lower flow type in the probing beam region.

From the FFoRM-SANS measurement of the CNC dispersion, we have extracted the direction of orientation and degree of alignment of a semi-dilute dispersion of CNC particles in the device. These results compare favorably to the asymptotic behavior of a model for dilute, rigid, rod-like dispersions in steady, 2D flows. The agreement between model and experiment suggests that, for the range of *Pe*_*r*_ and *Λ*_2*D*_ measured, the orientation behavior of this semi-dilute CNC dispersion can be captured by defining the flow in terms of the dimensionless applied rate of strain, *Pe*_r_, which accounts for both the non-dilute concentration as well as the flow type. Additionally, this agreement suggests that the deformations applied in the beam region are sufficiently uniform such that the measured structure is similar to the expected steady state structure in a homogeneous flow. Thus, we conclude that the microstructural response being measured by SANS in the device represents a near microstructural steady-state, with little or no Lagrangian variation through the beam region. We attribute the achievement of near steady-state microstructural configuration on the fact that there is a stagnation point within the scattering region, where convection out of the region of uniform flow is slowed (i.e. the accumulated strain is high) compared to ‘flow-through’ geometries such as contractions and expansions.

**Figure 14:**
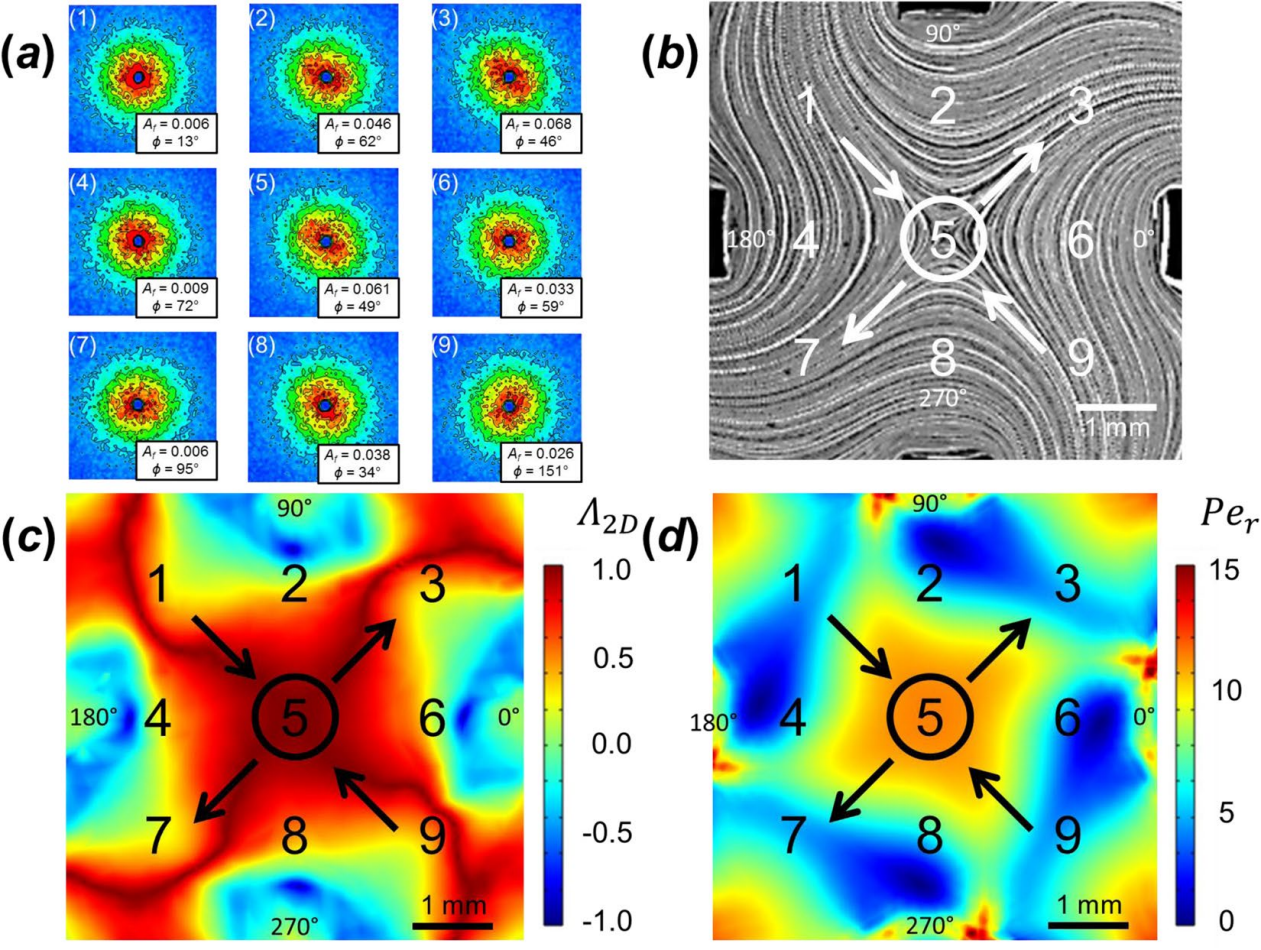
Flow field scan of CNC dispersion in FFoRM under extensional flow conditions (*Pe*_*r*_ = 10, 〈*Λ*_2*D*_〉 = 0.95). Numbered SANS spectra (**a**) correspond to the approximate location of the neutron beam (indicated with the D = 1 mm circles around the stagnation point region, 5) in the flow field (**b**) streaklines, (**c**) flow type parameter and (**d**) *Pe*_*r*_. Streaklines, flow type parameter, and *Pe*_*r*_ are shown at the center plane of the device and correspond to the experimental conditions of the SANS measurements with inflow and outflow directions indicated with arrows. Streaklines were generated from experiments while flow type parameter and *Pe*_*r*_ fields were generated from simulations. In the SANS spectra, blue color denotes low intensity while red denotes high intensity. Calculated *A*_*f*_ and *ϕ* are included underneath the corresponding spectra.

To illustrate the importance of the stagnation point, Fig. 14 compares flow-SANS measurements when the probing beam is placed at different points in the FFoRM device, including locations near the stagnation point with large accumulated strains as well as those further from the stagnation point where material is rapidly convected into and out of the scattering volume. SANS spectra (Fig. 14a) correspond to measurements at various locations, flow types, and *Pe*_*r*_ (Fig. 14b–d) within the FFoRM geometry. In this experiment we probed entry (1 and 9), exit (3 and 7), and transition (2, 4, 6, and 8) regions in addition to the previously reported stagnation point flow (5) under conditions corresponding to *Pe*_*r*_** = **10 and *Λ*_2*D*_ = 0.95 in the beam region. From these results, the impact of the Lagrangian unsteady nature of the flow outside the stagnation point, as well as non-uniformity of flow type within these regions, is made apparent. Specifically, in several spectra (1, 4, and 7), the microstructure shows no preferred alignment (*A*_*f*_ < 0.01) in any direction despite the presence of a sufficiently large local deformation rate (*Pe*_*r*_ > 1) for extension-dominated (*Λ*_2*D*_ > 0) flows in the beam region. We attribute this observed lack of alignment to the lack of a preferred orientation direction in the average orientation distribution function when averaging CNC orientations from different flow histories. One spectra (3) shows a very high degree of alignment, despite the lack of strong flows in the beam region. We attribute the increased alignment to the convection of highly aligned microstructure from near the stagnation point along the outflow axis. Due to the high velocities in this region, the microstructure does not have enough time to adopt a near steady-state orientation distribution before being convected out of the beam region. This result clearly illustrates the significance of the FFoRM’s ability to produce arbitrary flow types in the stagnation region of a nearly 2D flow for SANS measurements, as otherwise the interpretation of the resulting scattering anisotropy is convoluted by a number of effects including Lagrangian unsteady (i.e., history-dependent) responses of the fluid microstructure as well as large variations in flow type and deformation rate magnitude within the probing neutron beam.

## Conclusions

In this work, we have reported the first flow-SANS sample environment and associated measurement methods capable of probing complex fluid microstructure under near-2D steady state flows with a range of variable flow types within a single device, the fluidic four-roll mill (FFoRM). Modification of a previously designed microfluidic four-roll mill geometry using 2D CFD simulations resulted in the ability to produce relatively uniform, steady state flows of varying flow type in fluids with both Newtonian and some non-Newtonian rheological responses. In cases where the flow can be easily controlled, the modified FFoRM enables unambiguous measurement and interpretation of microstructural changes within a probing neutron beam. Inelastic fluids (shear thinning and yield stresses) generate stable, Newtonian-like flows while fluid elasticity generally modified flows from their nominal behavior beyond *Wi* = 1, requiring further characterization of the flow fields of elastic and viscoelastic fluids for FFoRM-SANS studies. The resulting FFoRM-SANS workflow was validated through measurement of a shear thinning, rod-like Brownian dispersion. Quantitative agreement between PTV measurements and flow simulations verify the achievable flows within the device, and demonstrate that simulation can be used to predict the mapping between device control variables and the resulting flow. FFoRM-SANS experiments confirmed that the device achieves near steady state, spatially uniform microstructural responses when the probing beam is placed in the stagnation region of the flow. Furthermore, the measurements on rod-like suspensions confirm a number of theoretical predictions including the preferred particle orientation and scaling of the degree of interparticle alignment with both the type and strength of the applied deformation. These results provide a small glimpse of the new scientific insights and structure-property relationships enabled by the ability of FFoRM-SANS to make microstructural measurements of fluids under controllably variable complex flows.

## Methods

### Materials

#### Newtonian fluid and solvents

Glycerol (Thermo Fisher, CAS # 56-81-5), de-ionized filtered water (Milli-Q), and deuterium oxide (Sigma-Aldrich, CAS # 7789-20-0) were used as received.

#### Inelastic shear thinning fluid

An aqueous dispersion of rod-like cellulose nanocrystals (University of Maine Process Development Center, Lot 2013-EPL-CNC-053) was used as a model shear thinning fluid with minimal elasticity. We prepared the sample by adding 5 wt% of the dry, white CNC powder to the solvent (10 wt% glycerol and 90 wt% D_2_O) and stirring overnight.

#### Yield stress fluid

Carbopol 934 (Lubrizol, CAS # 9003-01-4) was used as received and dissolved in water. The solution was then neutralized with 25 wt% potassium hydroxide (EM Science, CAS # 1310-58-3) solution by drop-wise addition. The final Carbopol concentration was 0.2 wt%.

#### Elastic (Boger) fluid

Polyethylene glycol (M_v_ = 8000 g/mol, Sigma-Aldrich, CAS # 25322-68-3) as received was dissolved in filtered water at 37.5 wt% and mixed for one hour. Polyethylene oxide (M_v_ = 4 × 10^6^ g/mol, Sigma-Aldrich) as received was then dissolved in this solution at 0.08 wt% and mixed for two weeks with a magnetic stir bar at low speed (60 rpm) to avoid polymer degradation.

#### Viscoelastic fluid

300 mM sodium nitrate (Sigma-Aldrich, CAS # 7631-99-4) and 100 mM hexadecyltrimetylammonium bromide (CTAB, Sigma-Aldrich, CAS # 5709-0) were used as received and dissolved in water, sonicated for 20 minutes, and allowed to rest for one day.

#### Rheology

The rheological tests were performed with an AR-G2 rheometer (TA Instruments) at constant temperature of 25 °C. To measure the rheological properties, steady-shear viscosity tests were performed using a Couette geometry for materials with low viscosity (cup diameter = 30.42 mm, truncation gap = 0.5 mm, and bob diameter and length of 27.95 mm and 42.15 mm, respectively) or a cone-plate geometry for materials with high viscosity and for normal stress measurements (diameter = 60 mm, angle = 2.00° and truncation gap = 0.055 mm). To prevent solvent evaporation, for the cone-plate, the material was thermally isolated with a solvent trap. Steady-shear viscosity tests were run in the range of shear rate 0.001 < $\dot{\gamma }$ <100 s^−1^. Data were collected once every 300 s, in order to reach the steady state.

#### Computational fluid dynamics (CFD) simulations

CFD simulations were carried out with the *COMSOL Multiphysics* software package for inelastic fliuds or in *OpenFOAM* using the viscoelasticFluidFoam solver for elastic fluids^39^. Flow fields for the inelastic fluids were generated from simulations for a Newtonian fluid (ρ = 1.261 g/mL, *η* = 1.412 Pa s) and a generalized Newtonian fluid with viscosity described by the Carreau model

10 $$\eta (\dot{\gamma })={\eta }_{0}{(1+{(\lambda \dot{\gamma })}^{2})}^{\frac{n-1}{2}}.$$

where *η*_0_ is the zero shear viscosity, *λ* is the relaxation time, and n is a parameter that determines the degree of shear thinning. Parameters were chosen as a fit of the steady shear rheology of the 5 wt% CNC dispersion, thus giving *η*_0_ = 2.51 Pa s, *λ* = 2.06 s, and n = 0.3. A critical shear rate can be defined as the inverse of the relaxation time (${\dot{\gamma }}_{critical}$ = 0.49 s^−1^) and corresponds to the onset of shear thinning. The generalized Navier-Stokes and continuity equations were solved numerically for a meshed representation of the geometry using a finite element method. Constant volume flow rate boundary conditions were specified at the ends of the channels corresponding to *Q*_1_ and *Q*_2_, constant pressure boundary conditions were specified at the ends of unlabeled channels, and no slip boundary conditions were specified for all other walls. Geometries were simulated in 3D when comparing to experimental data to ensure maximum accuracy and in 2D for geometry design to enable faster simulation times.

#### Fluidic four-roll mill (FFoRM) Fabrication

Devices consist of two outer plates squeezed around an inner plate containing the FFoRM geometry. The outer plates were constructed out of stainless steel and contain holes for the flow of temperature controlling fluid and holes for quartz windows to allow for light or neutrons to pass through the device. The inner plate was constructed out of titanium and the geometry was cut using a wire EDM. The central device geometry was cut according to the design in Fig. 1 and 35 mm channels were added as inlets/outlets into the center geometry to allow time for the fluid to relax from entry effects. Holes were drilled though the sides of the center plate, connecting to the eight entry channels, to allow for fluid flow straight into the device.

#### Particle tracking velocimetry (PTV)

Flows of the fluids in the FFoRM were experimentally determined using particle tracking velocimetry (PTV). The test fluid was seeded with glass spheres (10 μm diameter, 300 ppm) and injected into the device’s four inlet channels using two syringe pumps (pump: Harvard Apparatus PHD 2000, tubing: Saint-Gobain Versilon 2001) at different rates. The four additional outlets were immersed in a container of the test fluid. The outlet tubing was cut to similar length and immersed at similar height to avoid differences in pressure between outlet channels. The device temperature was maintained at 25 °C by using a water bath connected to the device’s outer plates. A CMOS camera (Point Gray Gazelle 2.2 MP Mono Camera Link) with 12x total magnification and variable frame rate (from 1 to 280fps) was focused on the center plane of the device geometry. The focal depth for the measurement is ~0.3 mm. Particle velocities were determined from videos using PTV algorithms that track individual particles with subpixel resolution and determine their velocities^29^. The velocity gradient tensor was calculated by fitting a second order surface using a weighted method of least squares^30,51^ adapted for 2D. The search radius is an adjustable parameter and the fit is weighted using the tri-cube kernel:

11 $$w(r)={(d-{|r|}^{3})}^{3}$$

where w is the weight a particular distance, r, from the point of interest and d is the distance one searches for velocity information. The local surface fit is differentiated to determine the components of the velocity gradient tensor. The process is repeated for the entire measured velocity field and results are binned to reduce numerical and experimental noise.

#### Flow-small-angle neutron scattering (flow-SANS)

SANS measurements were performed using the NGB 10 m SANS instrument at the National Institute of Standards and Technology Center for Neutron Research (Gaithersburg, MD). A 1 mm diameter beam was collimated at the stagnation point of the geometry. Material of interest was flowed into the device using the same protocols as for PTV experiments. Measurements were delayed for 5 minutes after startup to ensure the flow had reached steady state. The scattering from the sample was collected in the *q*-range from 0.005-0.1 Å^−1^ with the wavelength *λ* = 6Å and a wavelength spread of Δ*λ*/*λ* = 0.14. The scattering vector *q* is defined as *q = *4π sin(*θ*/2)/*λ* where *θ* is the angle at which the neutron is scattered and λ is the neutron wavelength. The temperature was maintained constant at 25 °C through the entire experiment with a water bath. The 2D scattering intensities in 128 by 128 channels were corrected for empty cell, plexiglass standard, and detector efficiency. Scattering spectra were reduced using standard NCNR protocols with Igor PRO software^52^.

## Electronic supplementary material

Supplementary Information

## Data Availability

The datasets generated during and/or analysed during the current study are available from the corresponding author on reasonable request.

## References

[1] Erb RM, Sander JS, Grisch R, Studart AR (2013). Self-shaping composites with programmable bioinspired microstructures. Nat. Commun..

[2] Sydney Gladman A, Matsumoto EA, Nuzzo RG, Mahadevan L, Lewis JA (2016). Biomimetic 4D printing. Nat. Mater..

[3] Wong HS (2014). The rheology and processing of “edge sheared” colloidal polymer opals. J. Rheol..

[4] Akbari A (2016). Large-area graphene-based nanofiltration membranes by shear alignment of discotic nematic liquid crystals of graphene oxide. Nat. Commun..

[5] Eberle APR, Porcar L (2012). Flow-SANS and Rheo-SANS applied to soft matter. Curr. Opin. Colloid Interface Sci..

[6] Lopez CG, Watanabe T, Martel A, Porcar L, Cabral JT (2015). Microfluidic-SANS: flow processing of complex fluids. Sci. Rep..

[7] Cinader DK, Burghardt WR (2000). Polydomain model predictions of liquid crystalline polymer orientation in mixed shear/extensional channel flows. Rheol. Acta.

[8] Clarke SM, Rennie AR, Convert P (2007). A diffraction technique to investigate the orientational alignment of anisotropic particles: studies of clay under flow. Europhys. Lett..

[9] Qazi SJS, Rennie AR, Wright JP, Cockcroft JK (2010). Alignment of plate-like particles in a colloidal dispersion under flow in a uniform pipe studied by high-energy x-ray diffraction. Langmuir.

[10] Penfold J (2006). Elongational flow induced ordering in surfactant micelles and mesophases. J. Phys. Chem. B.

[11] Penfold J, Tucker I (2007). Flow-induced effects in mixed surfactant mesophases. J. Phys. Chem. B.

[12] Kisilak M (2001). An x-ray extensional flow cell. Rev. Sci. Instrum..

[13] Idziak SHJ (2001). Undulating membrane structure under mixed extensional-shear flow. Eur. Phys. J. E.

[14] Bent J (2003). Neutron-Mapping Polymer Flow: Scattering, Flow Visualization, and Molecular Theory. Science.

[15] Graham RS (2006). Measuring and predicting the dynamics of linear monodisperse entangled polymers in rapid flow through an abrupt contraction. A small angle neutron scattering study. Macromolecules.

[16] Graham RS (2009). The long-chain dynamics in a model homopolymer blend under strong flow: small-angle neutron scattering and theory. Soft Matter.

[17] Burghardt WR, Brown EF, Auad ML, Kornfield JA (2005). Molecular orientation of a commercial thermotropic liquid crystalline polymer in simple shear and complex flow. Rheol. Acta.

[18] Taylor GII (1934). The Formation of Emulsions in Definable Fields of Flow. Proc. Roy. Soc..

[19] Fuller GG, Leal LG (1980). Flow birefringence of dilute polymer solutions in two-dimensional flows. Rheol. Acta.

[20] Fuller GG, Leal LG (1981). Flow Birefringence of Concentrated Polymer Solutions in Two-Dimensional Flows. J Polym Sci Polym Phys Ed.

[21] Lee JS, Dylla-Spears R, Teclemariam NP, Muller SJ (2007). Microfluidic four-roll mill for all flow types. Appl. Phys. Lett..

[22] Olbricht WL, Rallison JM, Leal LG (1982). Strong flow criteria based on microstructure deformation. J. Non-Newton. Fluid Mech..

[23] Woo NJ, Shaqfeh ESG (2003). The configurational phase transitions of flexible polymers in planar mixed flows near simple shear. J. Chem. Phys..

[24] Jain A, Sasmal C, Hartkamp R, Todd BD, Prakash JR (2015). Brownian dynamics simulations of planar mixed flows of polymer solutions at finite concentrations. Chem. Eng. Sci..

[25] Wagner CE, McKinley GH (2016). The importance of flow history in mixed shear and extensional flows. J. Non-Newton. Fluid Mech..

[26] Pakdel P, McKinley G (1996). Elastic Instability and Curved Streamlines. Phys. Rev. Lett..

[27] Gulati S, Dutcher CS, Liepmann D, Muller SJ (2010). Elastic secondary flows in sharp 90 degree micro-bends: A comparison of PEO and DNA solutions. J. Rheol..

[28] Soulages J (2008). Lubricated optical rheometer for the study of two-dimensional complex flows of polymer melts. J. Non-Newton. Fluid Mech..

[29] Crocker JC, Grier DG (1996). Methods of Digital Video Microscopy for Colloidal Studies. J. Colloid Interface Sci..

[30] Cheng P, Burroughs MC, Leal LG, Helgeson ME (2017). Distinguishing shear banding from shear thinning in flows with a shear stress gradient. Rheol. Acta.

[31] Habibi Y, Lucia LA, Rojas OJ (2010). Cellulose nanocrystals: Chemistry, self-assembly, and applications. Chem. Rev..

[32] Boluk Y, Zhao L, Incani V (2012). Dispersions of nanocrystalline cellulose in aqueous polymer solutions: Structure formation of colloidal rods. Langmuir.

[33] Haywood AD (2017). New insights into the flow and microstructural relaxation behavior of biphasic cellulose nanocrystal dispersions from RheoSANS. Soft Matter.

[34] Leal LG, Hinch EJ (1973). Theoretical studies of a suspension of rigid particles affected by Brownian couples. Rheol. Acta.

[35] Doi M, Edwards SF (1978). Dynamics of rod-like macromolecules in concentrated solution. Part 1. J. Chem. Soc. Faraday Trans. 2 Mol. Chem. Phys..

[36] Craig DQM, Tamburic S, Buckton G, Newton JM (1994). An investigation into the structure and properties of Carbopol 934 gels using dielectric spectroscopy and oscillatory rheometry. J. Control. release.

[37] Dontula P, Macosko CW, Scriven LE (1998). Model elastic liquids with water-soluble polymers. AIChE J..

[38] Jasak, H., Jemcov, A. & Tukovic, Z. OpenFOAM: A C++ Library for Complex Physics Simulations. *Int. Work. Coupled Methods Numer. Dyn*. 1–20 (2007).

[39] Favero JL, Secchi AR, Cardozo NSM, Jasak H (2010). Viscoelastic flow analysis using the software OpenFOAM and differential constitutive equations. J. Non-Newton. Fluid Mech..

[40] Dunlap PN, Leal LG (1987). Dilute polystyrene solutions in extensional flows: Birefringence and flow modification. J. Non-Newton. Fluid Mech..

[41] Boger DV (1987). Viscoelastic Flows Through Contractions. Ann. Rev. Fluid Mech..

[42] Arratia PE, Thomas CC, Diorio J, Gollub JP (2006). Elastic Instabilities of Polymer Solutions in Cross-Channel Flow. Phys. Rev. Lett..

[43] Helgeson ME, Hodgdon TK, Kaler EW, Wagner NJ (2010). A systematic study of equilibrium structure, thermodynamics, and rheology of aqueous CTAB/NaNO3 wormlike micelles. J. Colloid Interface Sci..

[44] Cates ME, Candau SJ (1990). Statics and dynamics of worm-like surfactant micelles. J. Phys. Condens. Matter.

[45] Hinch EJ, Leal LG (1976). Constitutive equations in suspension mechanics. Part 2. Approximate forms for a suspension of rigid particles affected by Brownian rotations. J. Fluid Mech..

[46] Mewis, J. & Wagner, N. J. *Colloidal suspension rheology*. *Colloidal Suspension Rheology* 9780521515, (Cambridge University Press, 2011).

[47] Haywood AD, Davis VA (2017). Effects of liquid crystalline and shear alignment on the optical properties of cellulose nanocrystal films. Cellulose.

[48] Walker Lynn M., Wagner Norman J. (1996). SANS Analysis of the Molecular Order in Poly(γ-benzyll-glutamate)/Deuterated Dimethylformamide (PBLG/d-DMF) under Shear and during Relaxation. Macromolecules.

[49] Helgeson ME, Vasquez PA, Kaler EW, Wagner NJ (2009). Rheology and spatially resolved structure of cetyltrimethylammonium bromide wormlike micelles through the shear banding transition. J. Rheol..

[50] Hinch EJ, Leal LG (1975). Constitutive equations in suspension mechanics. Part 1. General formulation. J. Fluid Mech..

[51] Cleveland WS, Devlin SJ (1988). Locally Weighted Regression: An Approach to Regression Analysis by Local Fitting. J. Am. Stat. Assoc..

[52] Kline SR (2006). Reduction and analysis of SANS and USANS data using IGOR Pro. J. Appl. Crystallogr..

