# Supplementary material for: Probing flow-induced nanostructure of complex fluids in arbitrary 2D flows using a fluidic four-roll mill (FFoRM)
Source: Sci Rep. 2018 Oct 22;8:15559. doi: 10.1038/s41598-018-33514-8 (PMC6197203; doi:10.1038/s41598-018-33514-8)
Supplement: Supplementary file 1 — Supplementary Information [file 41598_2018_33514_MOESM1_ESM.docx]

**Supplementary Materials: Probing flow-induced nanostructure of complex fluids in arbitrary 2D flows using a fluidic four-roll mill (FFoRM)**

**P. T. Corona, N. Ruocco, K. M. Weigandt, L. G. Leal and M. E. Helgeson**

*Section A: Approximations for steady flows of dilute rod-like solutions*

The development of the orientation distribution function, $\frac{\psi\left( \phi,\theta,t \right)}{\sin\left( \theta\right)}=N\left( \mathbf{p},t \right)$, for a homogeneous, dilute suspension of rigid particles with fore-aft symmetry at low *Re* is governed by

$\frac{DN}{Dt}+\frac{\partial}{\partial\mathbf{p}}\cdot\left( N\left( \boldsymbol{\Omega}\cdot\mathbf{p}+\frac{r^{2}-1}{r^{2}+1}\left[ \mathbf{E}\cdot\mathbf{p}-\left( \mathbf{p}\cdot\mathbf{E}\cdot\mathbf{p} \right)\mathbf{p} \right] \right) \right)=\frac{\partial}{\partial\mathbf{p}}\cdot\left( D_{r}\frac{\partial N}{\partial\mathbf{p}} \right)$ (S1)

where **p** is the particle orientation vector, **Ω** is the vorticity tensor, **E** is the rate of strain tensor, *r* is the particle aspect ratio, and *D_r_* is the particle’s rotational diffusivity.^1^ Furthermore, the distribution must be normalized such that $\int_{\mathbf{p}} N\left( \mathbf{p},t \right)d\mathbf{p}=1$. For the approximate 2D linear flows generated near the stagnation point in the fluidic four-roll mill (equation (2)), the rate of strain (**E**) and vorticity (**Ω**) tensors are given as

$\mathbf{E}=\frac{\dot{\Gamma}}{\sqrt{1+\Lambda^{2}}}\left[ \begin{matrix} 1+\Lambda& 0 & 0 \\ 0 & -\left( 1+\Lambda\right) & 0 \\ 0 & 0 & 0 \end{matrix} \right]$, $\boldsymbol{\Omega}=\frac{\dot{\Gamma}}{\sqrt{1+\Lambda^{2}}}\left[ \begin{matrix} 0 & 1-\Lambda& 0 \\ -\left( 1-\Lambda\right) & 0 & 0 \\ 0 & 0 & 0 \end{matrix} \right]$. (S2)

*Approximation in the weak flow limit*

Following Hinch and Leal (1976)^2^, in the limit of weak flows ($\frac{\left| \mathbf{E} \right|}{D_{r}}\boldsymbol{\equiv}{Pe}_{r}$<< 1), the asymptotic solution of equation S1 to first order approximation in *Pe_r_* is

$N=\frac{1}{4\pi}+{Pe}_{r}\frac{15}{8\pi}\left( \mathbf{p}\cdot\bar{\mathbf{A}}\cdot\mathbf{p} \right)+O\left( {{Pe}_{r}}^{2} \right)$ (S3)

where $\bar{\mathbf{A}}\boldsymbol{=}\frac{\mathbf{A}}{\left| \mathbf{E} \right|}$ is found for homogeneous flows in the limit of slowly varying, weak flows as

$\mathbf{A}=\frac{1}{15}\frac{r^{2}-1}{r^{2}+1}\mathbf{E}+O\left( {{Pe}_{r}}^{2} \right)$**.** (S4)

Substituting the velocity gradient tensor from equation (S2) into equation (S3) and equation (S4), the first order approximation for steady state orientation distribution in the weak flow limit becomes

$N=\frac{1}{4\pi}+{Pe}_{r}\frac{1}{8\sqrt{2}\pi}\frac{r^{2}-1}{r^{2}+1}\cos2\phi_{strain}\sin^{2} \theta+O\left( {{Pe}_{r}}^{2} \right)$ (S5)

where $\phi_{strain}$ is the in-plane angle with respect to the principle strain-rate axis (to distinguish it from the $\phi$ relative to the device geometry) and $\theta$ is the out-of-plane angle. Notably, the first deviation from an isotropic orientation distribution is a preferred orientation along the principle strain-rate axis, independent of the flow type. Furthermore, the magnitude of this first order correction is only a function of the *Pe_r_* and particle aspect ratio.

*Approximation in the strong flow limit*

In the limit of strong flows (i.e. negligible Brownian rotations), rigid, elongated particles will rotate along their Jeffrey’s orbits as a function of their initial orientation and velocity gradients encountered. Following Dinh and Armstrong (1984)^3^, for initially isotropic orientation distributions the orientation distribution is

$N=\frac{1}{4\pi}\left( {(\mathbf{B}^{-1})}^{T}\cdot\mathbf{B}^{-1}\dot{\cdot}\mathbf{pp} \right)^{-3/2}$ (S6)

where **B** is the deformation gradient $\left( B_{ij}=\frac{dx_{i}}{dx_{j}^{0}} \right)$ found for homogeneous flows as

$\mathbf{B}=e^{t \nabla\mathbf{u}}$ (S7)

Substituting the velocity gradient tensor from equation (S2) into equation (S6) and equation (S7) and in the limit of long times, the orientation distribution function with no Brownian diffusion becomes

$N=\frac{1}{4\pi}\left( e^{-\frac{4t\dot{\Gamma}\sqrt{\Lambda}}{\sqrt{1+\Lambda^{2}}}}\frac{\left( 1+\Lambda\right)}{8\Lambda}\sin^{2} \theta\left( \left( \sqrt{\Lambda}-1 \right)\cos\phi_{strain}+\left( \sqrt{\Lambda}+1 \right)\sin\phi_{strain} \right)^{2} \right)^{-3/2}.$ (S8)

From this orientation distribution function, the in-plane direction of maximum orientation is

$\phi_{strain, max}=\tan^{-1} \left( \frac{\sqrt{\Lambda}-1}{\sqrt{\Lambda}+1} \right)$. (S9)

This result gives the preferred direction of alignment in the strong flow limit (*Pe_r_* >> 1) relative to the principle strain-rate axis. This angle corresponds to the outflow axis of the flow. For strong flows $\left( \Lambda>0 \right)$ as $t\to\infty$ (i.e. steady state), the orientation distribution function becomes a delta function in this preferred alignment direction.

*Section B: Geometry modification operating diagrams*

In studies varying the geometric parameter *W* (see Figure 2 in the main text) while holding other parameters constant, we find a direct tradeoff between the maximum deformation rates achievable and the flow uniformity near the stagnation point. Devices with larger *W* generate flows that are more uniform, but with lower maximum deformation rates. The same monotonic trend is seen for the parameters *D* and *R*, albeit with less impact on the flow than changes in *W*. We chose to fix *D* = 1 mm because we found saturation in the flow homogeneity when the size of *D* was increased to the size of the desired homogeneous flow region. We chose to fix *R* = 1 mm because we found that changes in *R* had very little impact on the overall flow below this value. This choice was made to accommodate flows of elastic and viscoelastic fluids in the device for which the flow stability is highly sensitive to sharp corners.^4^ The results for variation of the geometric parameters *W*, *D*, and *R* are included in the Supplementary Materials. Studies varying the geometric parameter *C* with *W*, *D*, and *R* fixed show a non-monotonic dependence of flow homogeneity with size and are discussed in more detail in the next paragraph.


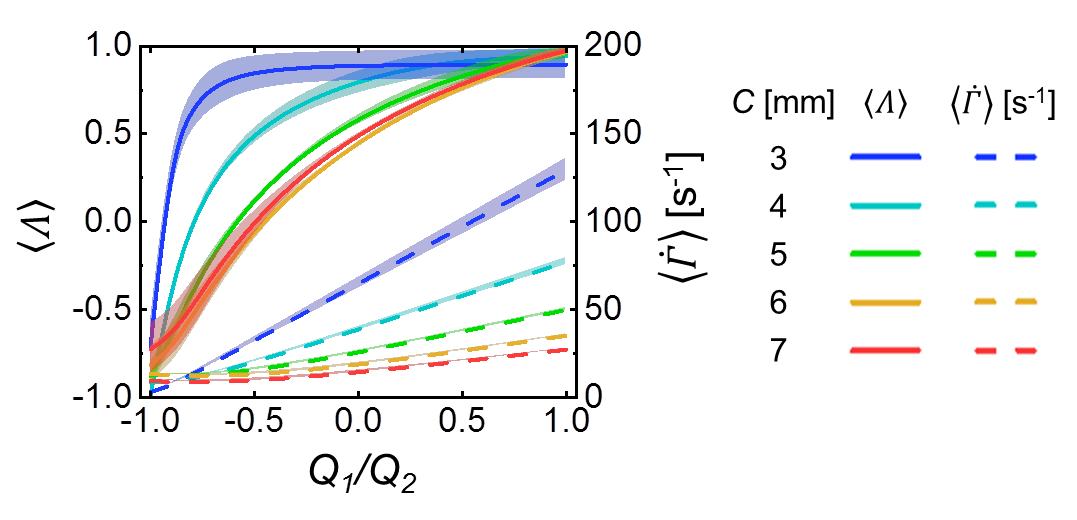


**Figure S1.** Representative results for 2D FFoRM operating diagrams for the flow of a Newtonian fluid (*ρ* = 1.261 g/mL and $\eta$ = 1.412 Pa s) while varying the geometric parameter *C* (see Figure 2 in the main text). The average magnitude of the velocity gradient tensor $\left\langle\dot{\Gamma} \right\rangle$ (dotted lines) and average flow type parameter $\left\langle\Lambda\right\rangle$ (solid lines) are plotted as a function of the operating condition *Q_1_/Q_2_* and the geometric parameter *C* for geometries with *W* = 2 mm, *D* = 1 mm, *R* = 1 mm, and *H* = 3 mm. Shaded areas around the average flow type parameter and magnitude of velocity gradient tensor represent the standard deviation of the respective value in the central 1 mm neutron beam region.

Figure S1 shows variations in $\left\langle\Lambda\right\rangle$, $\sigma_{\Lambda}$, $\left\langle\dot{\Gamma} \right\rangle$, and $\sigma_{\dot{\Gamma}}$ upon varying the geometric parameter *C* (see Figure 2 in the main text) and holding all other geometric parameters fixed. Qualitatively, we find that geometries with larger values of *C* generate strain dominated flows ($\left\langle\Lambda\right\rangle$ > 0) that are more homogeneous while, unlike other geometric parameters, smaller values of *C* generate vorticity dominated flows ($\left\langle\Lambda\right\rangle$ < 0) that are more uniform (as indicated by $\sigma_{\Lambda}$). These competing effects lead to an optimal size to produce homogeneous flows for all flow types where *C* = 2*W* + *D*. Physically, this means that the optimal length that the channel dividers extend into the central region is such that they are in line with the entry channel’s outer boundary. As with the other geometric parameters, smaller values of *C* generate flows with higher $\left\langle\dot{\Gamma} \right\rangle$ for all *Q_1_/Q_2_*. In this example situation, we find that the geometries where *C* = 5 mm and *C* = 6 mm are capable of producing sufficiently uniform flows for all flow types, but a value of *C* = 5 mm enables the generation of maximum deformation rates that are approximately 50% higher than for *C* = 6 mm.

As an aside, an important consideration for the design of the FFoRM is the stability of flows to fluctuations in flow rates into the device. Fluctuations in the ratio *Q_1_/Q_2_* can have a profound impact on stability in $\left\langle\Lambda\right\rangle$ in FFoRM designs. In Figure S1, the design with *C* = 3 mm (blue) shows a steep slope in the *Q_1_*/*Q_2_* vs. $\left\langle\Lambda\right\rangle$ curve near *Q_1_*/*Q_2_* = -1, meaning that small changes in *Q_1_*/*Q_2_* in this region can vary $\left\langle\Lambda\right\rangle$ drastically. While the slope of the *Q_1_*/*Q_2_* vs. $\left\langle\Lambda\right\rangle$ curve was not directly accounted for in this work, future device designs should ensure that the slope remains as linear as possible over the operating range of interest to avoid high device sensitivity to fluctuations in inlet flow rates.

The results for varying geometric parameters *W*, *D*, and *R* are included holding the non-varied parameters constant at *W* = 2 mm, *D* = 1 mm, *C* = 5 mm, and *R* = 1 mm.

*Channel width (W)*

Figure S2 shows variations in $\left\langle\Lambda\right\rangle$, $\sigma_{\Lambda}$, $\left\langle\dot{\Gamma} \right\rangle$, and $\sigma_{\dot{\Gamma}}$ upon varying the geometric parameter *W* and holding all other geometric parameters fixed.


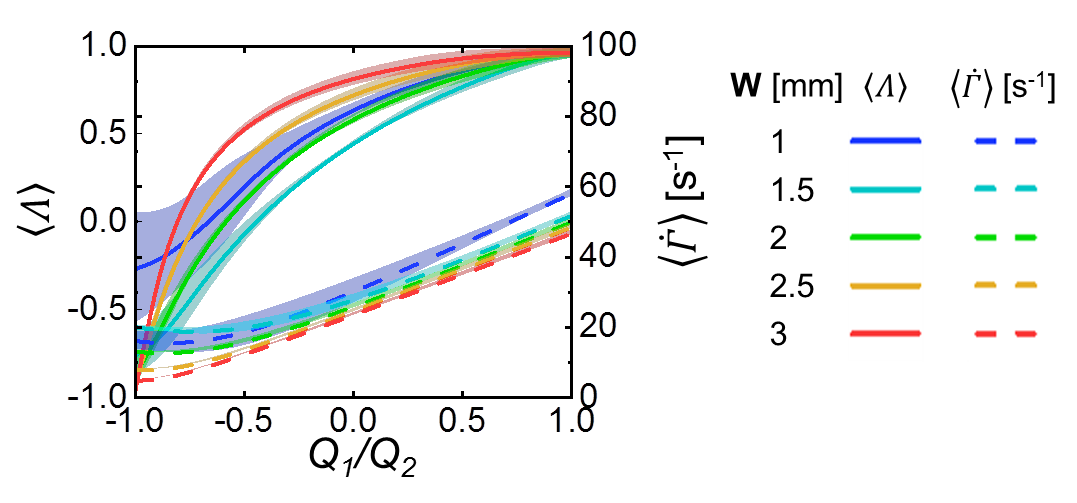


**Figure S2.** 2D FFoRM operating diagrams for the flow of a Newtonian fluid (*ρ* = 1.261 g/mL and $\eta$ = 1.412 Pa s) while varying the geometric parameter *W* (see Figure 2 in the main text). The average magnitude of the velocity gradient tensor $\left\langle\dot{\Gamma} \right\rangle$ (dotted lines) and average flow type parameter $\left\langle\Lambda\right\rangle$ (solid lines) are plotted as a function of the operating condition *Q_1_/Q_2_* and the geometric parameter *W* for geometries with *C* = 5 mm, *D* = 1 mm, *R* = 1 mm, and *H* = 3 mm. Shaded areas around the average flow type parameter and magnitude of velocity gradient tensor represent the standard deviation of the respective value in the central 1 mm region.

*Channel divider width (D)*

Figure S3 shows variations in $\left\langle\Lambda\right\rangle$, $\sigma_{\Lambda}$, $\left\langle\dot{\Gamma} \right\rangle$, and $\sigma_{\dot{\Gamma}}$ upon varying the geometric parameter *D* and holding all other geometric parameters fixed.


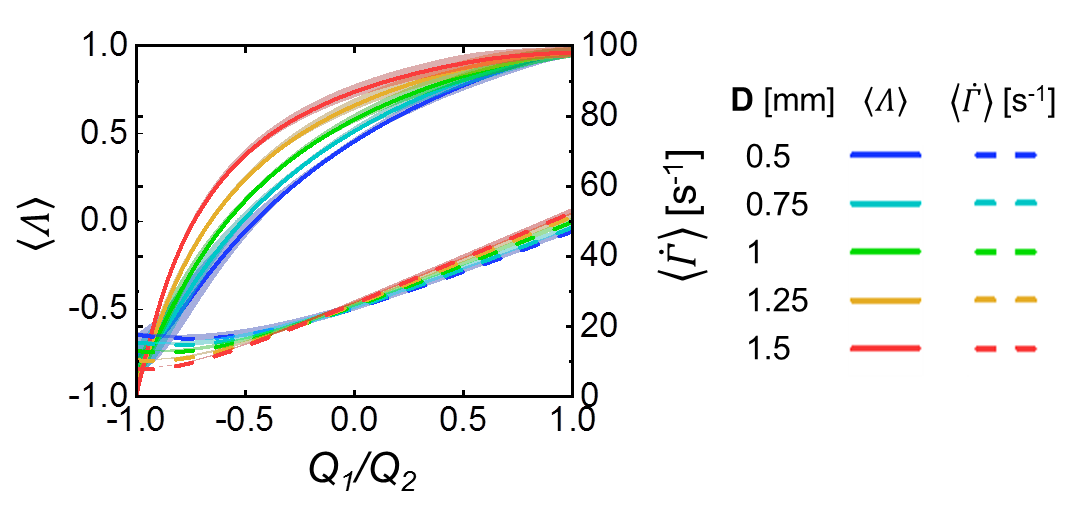


**Figure S3.** 2D FFoRM operating diagrams for the flow of a Newtonian fluid (*ρ* = 1.261 g/mL and $\eta$ = 1.412 Pa s) while varying the geometric parameter *D* (see Figure 2 in the main text). The average magnitude of the velocity gradient tensor $\left\langle\dot{\Gamma} \right\rangle$ (dotted lines) and average flow type parameter $\left\langle\Lambda\right\rangle$ (solid lines) are plotted as a function of the operating condition *Q_1_/Q_2_* and the geometric parameter *D* for geometries with *C* = 5 mm, *W* = 2 mm, *R* = 1 mm, and *H* = 3 mm. Shaded areas around the average flow type parameter and magnitude of velocity gradient tensor represent the standard deviation of the respective value in the central 1 mm region.

*Corner radius of curvature (R)*

Figure S4 shows variations in $\left\langle\Lambda\right\rangle$, $\sigma_{\Lambda}$, $\left\langle\dot{\Gamma} \right\rangle$, and $\sigma_{\dot{\Gamma}}$ upon varying the geometric parameter *R* and holding all other geometric parameters fixed.


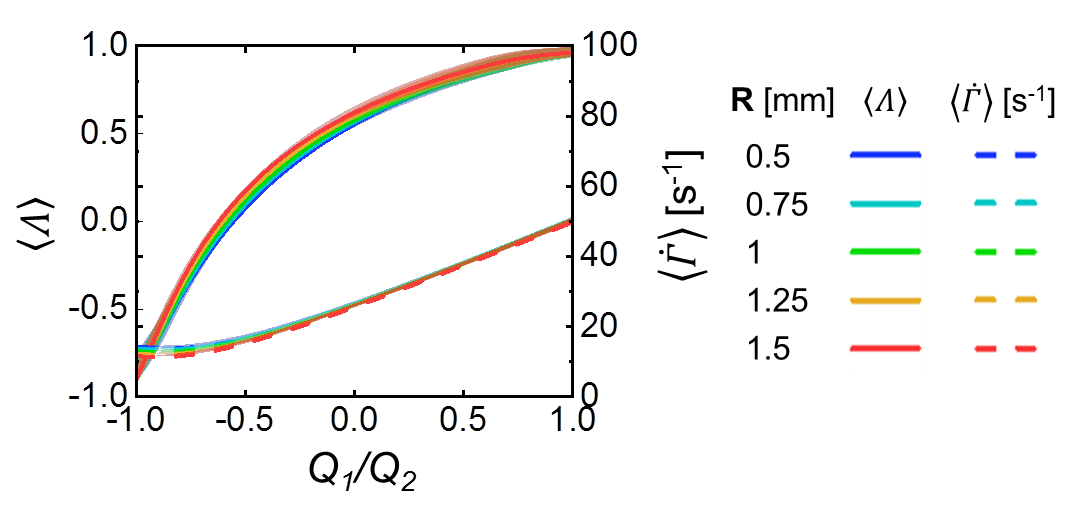


**Figure S4.** 2D FFoRM operating diagrams for the flow of a Newtonian fluid (*ρ* = 1.261 g/mL and $\eta$ = 1.412 Pa s) while varying the geometric parameter *R* (see Figure 2 in the main text). The average magnitude of the velocity gradient tensor $\left\langle\dot{\Gamma} \right\rangle$ (dotted lines) and average flow type parameter $\left\langle\Lambda\right\rangle$ (solid lines) are plotted as a function of the operating condition *Q_1_/Q_2_* and the geometric parameter *R* for geometries with *C* = 5 mm, *W* = 2 mm, *D* = 1 mm, and *H* = 3 mm. Shaded areas around the average flow type parameter and magnitude of velocity gradient tensor represent the standard deviation of the respective value in the central 1 mm region.

*Section C: Accumulated strain and 3D flow analysis*

To calculate the accumulated strain at various points in the measurement region of the FFoRM, we begin by assuming the beam region represents a region of homogeneous, 2D linear flow (equation (2) in the main text with velocities equal to zero at the stagnation point). Furthermore, we assume no migration of fluid elements across streamlines. One may calculate the accumulated strain (*ε*) along a streamline in the beam region as

$\varepsilon\left( x_{1},x_{2} \right)=\left| \mathbf{E} \right|t=\frac{\dot{\Gamma}}{2\sqrt{1+\Lambda^{2}}}\left( \Lambda+1 \right) t\left( x_{1},x_{2} \right)$ (S10)

where *t* is the residence time in the region of homogeneous flow. The residence time along a streamline can be calculated as

$t\left( x_{1},x_{2} \right)=\int\frac{ds}{\left| \boldsymbol{u}(s) \right|}= \int_{x_{1,entry}}^{x_{1}} dx_{1}\frac{\sqrt{1+\left( \frac{dx_{2}}{dx_{1}}|_{\Psi(x_{1},x_{2})} \right)^{2}}}{\sqrt{{u_{1}(x_{1},x_{2})}^{2}+{u_{2}(x_{1},x_{2})}^{2}}}$ (S11)

where *x*_1_ and *x*_2_ are the coordinates for the position in the beam region, *x*_1,entry_ is the position of entry into the beam region, $\Psi(x_{1},x_{2})$ is the value of the streamfunction, and *u*_1_(*x*_1_,*x*_2_) and *u*_2_(*x*_1_,*x*_2_) are the velocities in the 1 and 2 direction respectively. The streamfunction can be solved for by integrating equation (2) in the main text. The velocities and position of entry into the beam can be determined from this streamfunction. Equation (S10) and equation (S11) are solved with numerical integration to determine the accumulated strain at points within the region of homogeneous flow. Notably, the accumulated strain does not depend on the deformation rate magnitude, only the size of the region of homogeneous flow and flow type.

To quantify the degree to which out of plane gradients exist in the region of the device being probed by the neutron beam, we utilized previously described 3D simulations of a Newtonian fluid in the FFoRM. Figure S5 includes representative results for the in-plane magnitude of the rate-of-strain tensor variation in the out-of-plane (z) direction of the device.

**
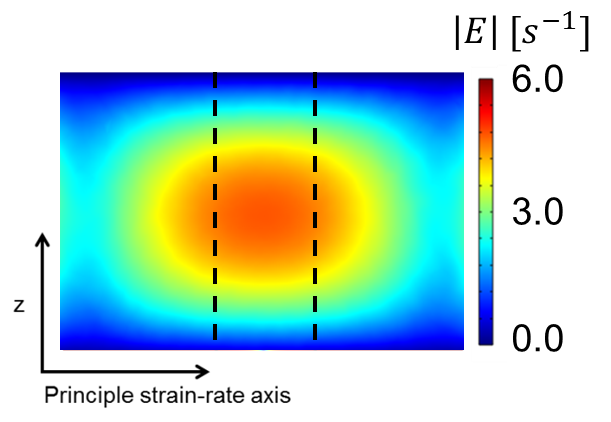
**

**Figure S5.**Magnitude of the in-plane components of the rate-of-strain tensor in the z-x plane of the center of the FFoRM geometry. Results are for a Newtonian fluid in the FFoRM geometry with *Q_1_*/*Q_2_* = 1.0 and *Q_2_* = 5.0 mL/min. Dotted lines indicate the bounding diameter of the probing neutron beam.

In addition to the variation in strain-rate through the thickness of the FFoRM geometry, we are also concerned with the fraction of the magnitude of the velocity gradient tensor that results from out of plane gradients (components including *u_3_*=w or z). Figure S6 includes the magnitude of the out-of-plane gradients divided by the magnitude of all gradients.

**
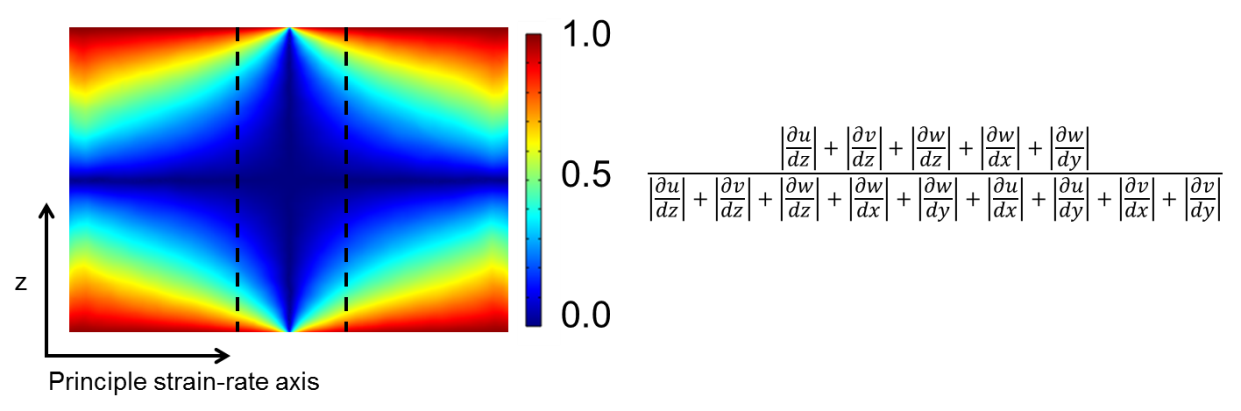
**

**Figure S6.**Fraction of total velocity gradient tensor magnitude that is out of plane in the 3D FFoRM geometry. Results are for a Newtonian fluid in the FFoRM geometry with *Q_1_*/*Q_2_* = 1.0 and *Q_2_* = 5.0 mL/min. Dotted lines indicate the bounding diameter of the probing neutron beam.

*Section D: Modified FFoRM geometry with rounded features*

For the flows of the elastic (Boger) fluid and viscoelastic fluid, a slightly modified geometry was employed to avoid flow modification due to the presence of sharp corners typical in fluids with significant elasticity.^4^ This modified geometry shape is included in Figure S7. Figure S7 also includes representative streakline images and local 2D flow type parameter for the flow of the CTAB/NaNO_3_ viscoelastic wormlike micelle solution at *Wi* < 0.1 where the fluid behavior is approximately Newtonian. This new geometry does not significantly alter the flows of Newtonian fluids in the FFoRM device as compared to the sharp cornered device (Figure 4 in the main text).


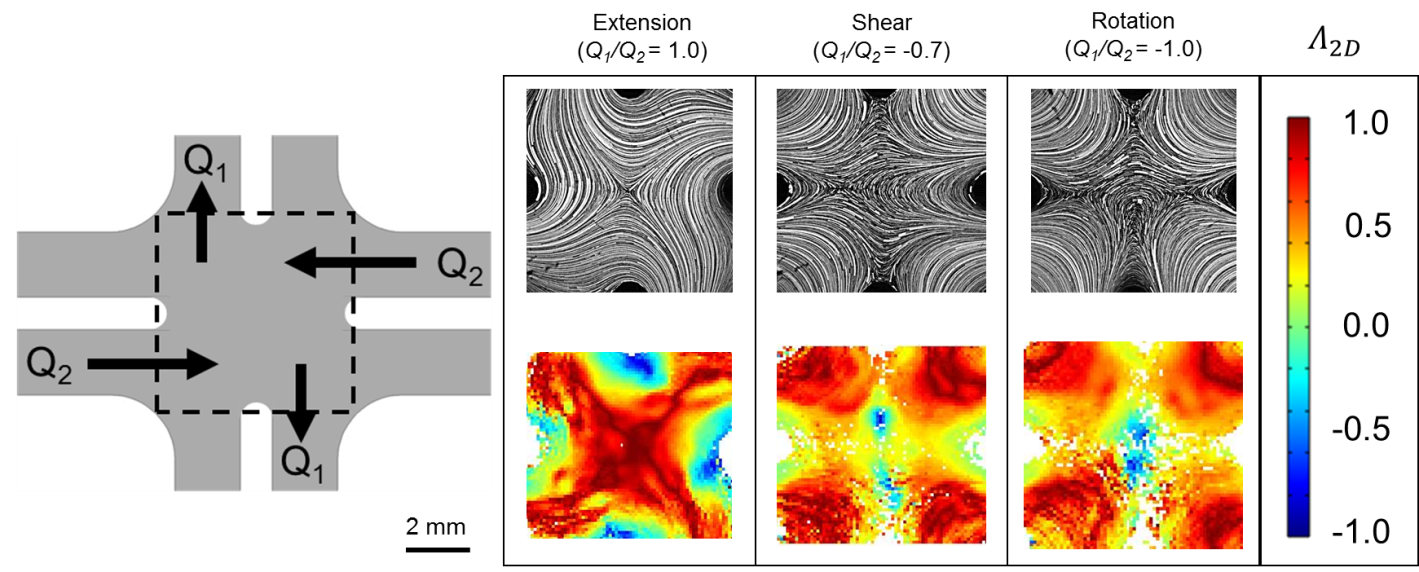


**Figure S7.** Streaklines (upper) and local 2D flow type parameter (*Λ_2D_*, lower) for the flow of CTAB/NaNO_3_ wormlike micelles (*Wi* < 0.02) in the center plane of the FFoRM geometry. All tests correspond to conditions with constant *Q_2_* = 0.005 mL/min (*Re* = 0.00025) and the values of *Q_1_/Q_2_* indicated. Experimental conditions correspond to 〈$\dot{\Gamma}$〉 ~0.05 s^-1^ for extension, ~0.025 s^-1^ for shear, and ~0.02 s^-1^ for rotation. The device geometry is included on the left with the region of interest for PTV experiments outlined for reference.

**References:**

1. Hinch, E. J. & Leal, L. G. Constitutive equations in suspension mechanics. Part 1. General formulation. *J. Fluid Mech.* **71,** 481–495 (1975).

2. Hinch, E. J. & Leal, L. G. Constitutive equations in suspension mechanics. Part 2. Approximate forms for a suspension of rigid particles affected by Brownian rotations. *J. Fluid Mech.* **76,** 187–208 (1976).

3. Dinh, S. M. & Armstrong, R. C. A Rheological Equation of State for Semiconcentrated Fiber Suspensions. *J. Rheol.* **28,** 207–227 (1984).

4. Pakdel, P. & McKinley, G. Elastic Instability and Curved Streamlines. *Phys. Rev. Lett.* **77,** 2459–2462 (1996).
